# Impact of the thyroid hormone T3 and its nuclear receptor TRα1 on colon cancer stem cell phenotypes and response to chemotherapies

**DOI:** 10.1038/s41419-024-06690-x

**Published:** 2024-05-01

**Authors:** Maria Virginia Giolito, Serguei Bodoirat, Theo La Rosa, Mathieu Reslinger, Gabriela D. A. Guardia, Jana Mourtada, Leo Claret, Alain Joung, Pedro A. F. Galante, Luiz O. F. Penalva, Michelina Plateroti

**Affiliations:** 1 Université de Strasbourg, INSERM, IRFAC/UMR-S1113, FMTS, 67200 Strasbourg, France; 2 Stem-Cell and Brain Research Institute, U1208 INSERM, USC1361 INRA, 69675 Bron, France; 3 Université de Strasbourg, CNRS, INSERM, IGBMC UMR 7104-UMR-S 1258, Illkirch, France; 4 Centro de Oncologia Molecular, Hospital Sírio-Libanês, São Paulo, Brazil; 5 Laboratoire de Biologie Tumorale, Institut de Cancérologie Strasbourg Europe, Strasbourg, France; 6 Greehey Children’s Cancer Research Institute, University of Texas Health Science Center at San Antonio, San Antonio, TX USA; 7 Present Address: Pole of Pharmacology and Therapeutics (FATH), Institut de Recherche Experimentale et Clinique (IREC), UCLouvain, Avenue Hippocrate 57, B1.57.04, B-1200 Brussels, Belgium

**Keywords:** Cancer stem cells, Cancer models, Gastrointestinal cancer

## Abstract

Colorectal cancers (CRCs) are highly heterogeneous and show a hierarchical organization, with cancer stem cells (CSCs) responsible for tumor development, maintenance, and drug resistance. Our previous studies showed the importance of thyroid hormone-dependent signaling on intestinal tumor development and progression through action on stem cells. These results have a translational value, given that the thyroid hormone nuclear receptor TRα1 is upregulated in human CRCs, including in the molecular subtypes associated with CSC features. We used an established spheroid model generated from the human colon adenocarcinoma cell line Caco2 to study the effects of T3 and TRα1 on spheroid formation, growth, and response to conventional chemotherapies. Our results show that T3 treatment and/or increased TRα1 expression in spheroids impaired the response to FOLFIRI and conferred a survival advantage. This was achieved by stimulating drug detoxification pathways and increasing ALDH1A1-expressing cells, including CSCs, within spheroids. These results suggest that clinical evaluation of the thyroid axis and assessing TRα1 levels in CRCs could help to select optimal therapeutic regimens for patients with CRC.

**Figure:**
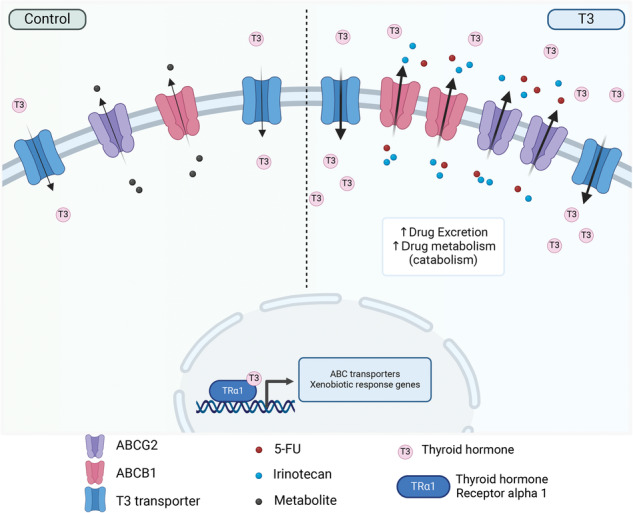
Proposed mechanism of action of T3/TRα1 in colon cancer spheroids. In the control condition, TRα1 participates in maintaining homeostatic cell conditions. The presence of T3 in the culture medium activates TRα1 action on target genes, including the drug efflux pumps *ABCG2* and *ABCB1*. In the case of chemotherapy FOLFIRI, the increased expression of ABC transcripts and proteins induced by T3 treatment is responsible for the augmented efflux of 5-FU and Irinotecan from the cancer cells. Taken together, these mechanisms contribute to the decreased efficacy of the chemotherapy and allow cells to escape the treatment. Created with BioRender.com.

Proposed mechanism of action of T3/TRα1 in colon cancer spheroids. In the control condition, TRα1 participates in maintaining homeostatic cell conditions. The presence of T3 in the culture medium activates TRα1 action on target genes, including the drug efflux pumps *ABCG2* and *ABCB1*. In the case of chemotherapy FOLFIRI, the increased expression of ABC transcripts and proteins induced by T3 treatment is responsible for the augmented efflux of 5-FU and Irinotecan from the cancer cells. Taken together, these mechanisms contribute to the decreased efficacy of the chemotherapy and allow cells to escape the treatment. Created with BioRender.com.

## Introduction

Colorectal cancer (CRC) is the second-leading cause of cancer death worldwide [1]. CRC results from a progressive acquisition and accumulation of genetic mutations and epigenetic alterations [2, 3], including oncogenic activation and inactivation of tumor suppressor genes [3, 4]. Moreover, non-genetic factors (e.g., the microenvironment) can promote oncogenic transformation and development of CRCs [5].

CRCs are heterogeneous and complex tumors. Their cell hierarchy, including undifferentiated cancer stem cells (CSCs) and differentiated tumor-bulk cells, resembles crypt organization in the normal colon [6, 7]. CSCs are a dynamic population continuously shaped by a convergence of genetic, epigenetic, and microenvironmental factors [6, 8]. They are responsible for tumor initiation [9], maintenance and growth, and metastatic capacity [10]. Moreover, CSCs are likely the main contributors to therapeutic resistance [6, 7]. Therefore, a better understanding of how CSCs acquire resistance to therapy is of fundamental importance.

Thyroid dysfunction is associated with several types of cancer, including CRC [11]. Although epidemiologic studies on the involvement of thyroid hormones (TH) in CRC have conflicted, most point towards hyperthyroidism and TH supplementation as predisposing risk factors for CRC (rev. in [12–16]). Our laboratory has contributed to the field by studying the function of the THs and their nuclear hormone receptor TRα1 in intestinal physiology and CRC. The targeted expression of TRα1 in the mouse intestinal epithelium in an *Apc*-mutated background is responsible for accelerating tumor appearance, progression, and aggressiveness compared to the simple *Apc* mutants [17]. Additional work demonstrated, for the first time, upregulation of the *THRA* gene and TRα1 receptor in human CRCs and a direct correlation between TRα1 and the Wnt pathway in this same context [18, 19]. However, few studies have described the involvement of THs and TRα1 in response to cancer treatment.

Resistance to chemotherapeutic treatments in CRC contributes to poor outcomes. Since the late 50s, 5-fluorouracil (5-FU) has constituted the backbone of CRC chemotherapeutic regimens. Response to 5-FU as a single agent is limited, and fewer than one-third of patients respond. However, adding 5-FU to oxaliplatin- or irinotecan-based therapies (FOLFOX and FOLFIRI, respectively) ameliorates the response rate by 50% [20]. Additionally, targeted therapies like cetuximab and panitumumab alone are effective only in approximately 10% of cases but are more successful when combined with classical chemotherapy. Nevertheless, resistance to all treatments has been observed repeatedly in CRC [20, 21]. Resistance to FOLFOX and FOLFIRI can be due to several mechanisms, including alteration of drug metabolism, detoxification, DNA repair, and adaptation to stressful conditions [21–24]. Multidrug resistance is considered one of the major causes of chemotherapy failure. It is often associated with the overexpression of ATP-binding cassette (ABC) transporter proteins, such as ABCB1 and ABCG2 [25].

Many genes altered in response to treatments, including ABC transporters, are regulated by the THs and/or their receptor TRs in the intestine or other organs [26–29]. These observations and the already demonstrated action of THs and/or TRs in intestinal stem cells and CRCs prompted us to analyze their role in tumor cells, including CSC biology. Using a spheroid model we developed [30], we evaluated the roles of TH and altered TRα1 levels in spheroid development and their response to FOLFOX and FOLFIRI. We observed that T3 treatment or TRα1 upregulation facilitated spheroid formation and growth and conferred a differential response to FOLFIRI, resulting in a resistant phenotype.

## Results

### Effects of T3 on spheroid formation and growth

**Fig. 1:**
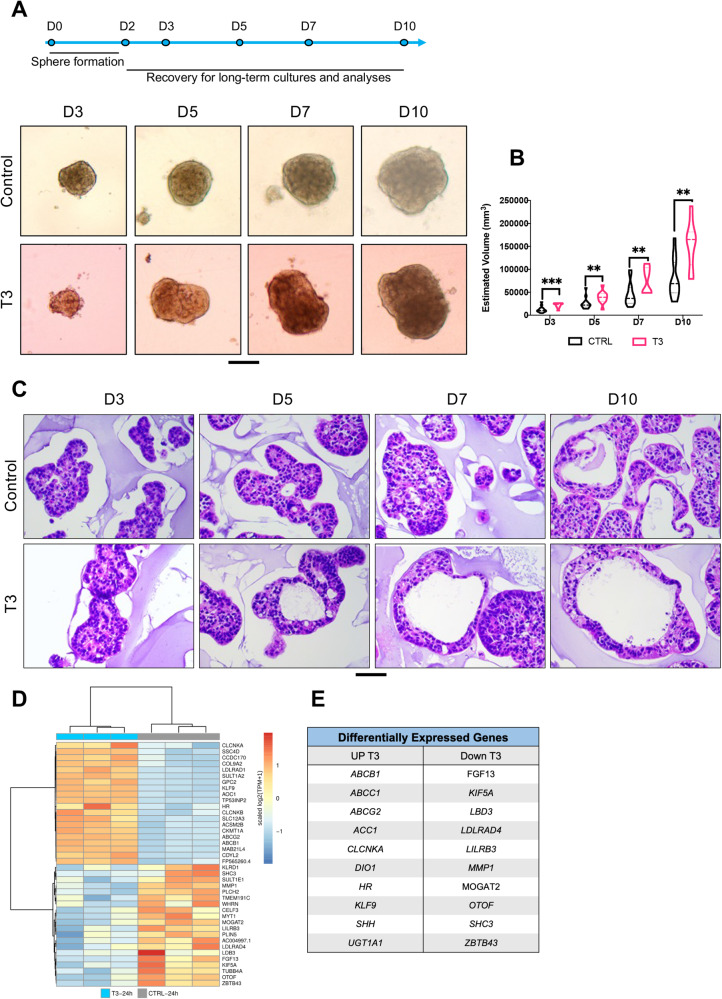
Effects of T3 on spheroid formation and growth. **A** Upper panel. Scheme of the experimental set-up and timeline. Lower panel. Representative pictures at each time point of spheroid cultures in different conditions as indicated. Images were taken under a Zeiss AxioVert microscope with a 4X objective. Scale bar: 200 μm. **B** Estimated volume of the spheroids in the different culture conditions at different time points, as indicated. Violin plots show the frequency distribution of the data; bold dotted lines indicate the median and light dotted lines indicate the quartiles, *n* = 16. Black spheres indicate the size of individual spheroids. **P < 0.01 and ***P < 0.001 compared to control (CTRL) condition, by multiple unpaired, two-tailed Student *t*-test. Results are representative of two independent experiments. **C** H&E staining of paraffin sections. Representative images of spheroids at the indicated time points after harvesting. Scale bar: low magnification: 100 μm; high magnifications: 50 μm. **D**, **E** Results of comparative RNA-seq transcription profile analyses of spheroids treated with T3 for 24 h during spheroid formation. Heat map (**D**) shows the 20 most upregulated or downregulated genes in T3 *vs.* control condition. **E** Selected genes differentially expressed between conditions.

To study the effects of the hormone T3 on Caco2-derived spheroids, we compared non-treated controls with cultures treated with 10^-7^M T3 for 48 h during spheroid formation. Spheroids were recovered and transferred to agarose-coated plates, and their volume was measured over time, using a specific formula [30, 31] (experimental plan summarized in Fig. 1A, upper panel). Independently from the condition, the volume increased in size during culture time but T3-treated spheroids had a significantly higher volume than the control spheroids at all time points (Fig. 1A, B).

We recovered the spheroids at different time points for detailed histologic and molecular analyses. Hematoxylin and eosin (H&E) staining showed that the spheroids changed their organization and shape over time. Both control and T3 spheroids presented cells densely arranged in multilayers at D3, while at D10, several zones were arranged in monolayers. Also, in both cases, a lumen appeared within the spheroids from D5 onwards, but the lumens were larger in T3 spheroids than in controls (Fig. 1B, C). After D10, the growth lowered and reached a plateau (our unpublished observations).

We performed RNA-seq analyses to elucidate the molecular events induced by brief T3 treatment. Spheroids were allowed to form for 48 h, T3 was added during the last 24 h of culture, and spheroids were collected for RNA-seq. Compared to controls, T3 spheroids had 227 upregulated and 67 downregulated genes (log2 fold change > 0.5, *P* value < 0.05) (Fig. 1D, Table S3). Among the upregulated genes (Fig. 1D, E) were known T3 target genes such as *KLF9* and *HR* [32, 33], *DIO1*, which is linked to TH metabolism [34, 35], and several ABC transporters. Furthermore, several downregulated genes were linked to cell signaling through FGF (*FGF13*) or TGFβ (*LDLRAD4*), lipid metabolism (*MOGAT2*), or calcium ion sensing (*OTOF*) (Fig. 1D, E).

**Fig. 2:**
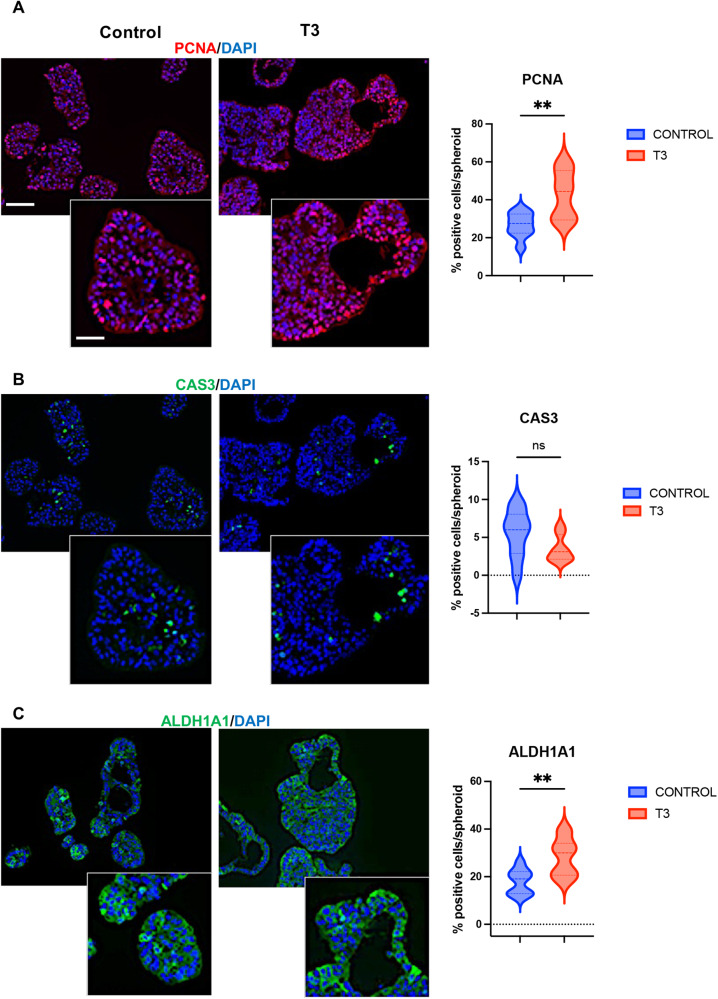
Characterization of T3-treated spheroids by immunolabeling. Immunostaining of spheroids at D7 for markers of proliferation: PCNA (**A**), cell death, Activated-caspase 3 (CAS3) (**B**), and CSC-like ALDH1A1 (**C**) in different conditions. Left panels: merging between each specific labeling and DAPI (nuclei, blue). Results shown are representative of at least 3 independent experiments. Images were taken with a 20× objective under a Zeiss AxioImager M2 Apotome 2 microscope. Scale bar: low magnification 50 μm, high magnification 25 μm. Right panels: percentage of positive cells per organoid for each marker in the different conditions. Violin plots show the frequency distribution of the data. Bold dotted lines indicate the median, and light dotted lines indicate the quartiles. *n* = 10 spheroids *per* marker and condition. ns not significant and **P < 0.01 compared to the control condition, by unpaired, two-tailed Student *t*-test. Number of cells scored: PCNA and CAS3, Control 726 and T3 1273; ALDH1A1, Control 432 and T3 1506.

Next, we analyzed the impact of T3 treatment on cell proliferation, cell death, and the presence of CSCs by immunolabeling (Fig. 2). For these analyses, we focused on D7 when the growth is active. Proliferating PCNA-positive cells were located at the external surface of the spheroids, sometimes in crypt-like or bud-like structures reminiscent of organoid cultures [36] (Fig. 2A, left panel). In the T3 condition, there was a statistically significant increase in PCNA-positive cells compared to controls (Fig. 2A, right panel). Surprisingly, we observed very few activated-CAS3-positive apoptotic cells in control or T3-treated spheroids, and the percentages were similar across groups (Fig. 2B). ALDH1A1-positive cells, possibly CSCs, were present throughout spheroids but were more frequently located on the external surface. They were organized in patches, and expression levels varied among cells, independently of the experimental condition (Fig. 2C, left panel). The percentage of ALDH1A1-expressing cells, however, was significantly increased by T3 treatment (Fig. 2C, right panel).

**Fig. 3:**
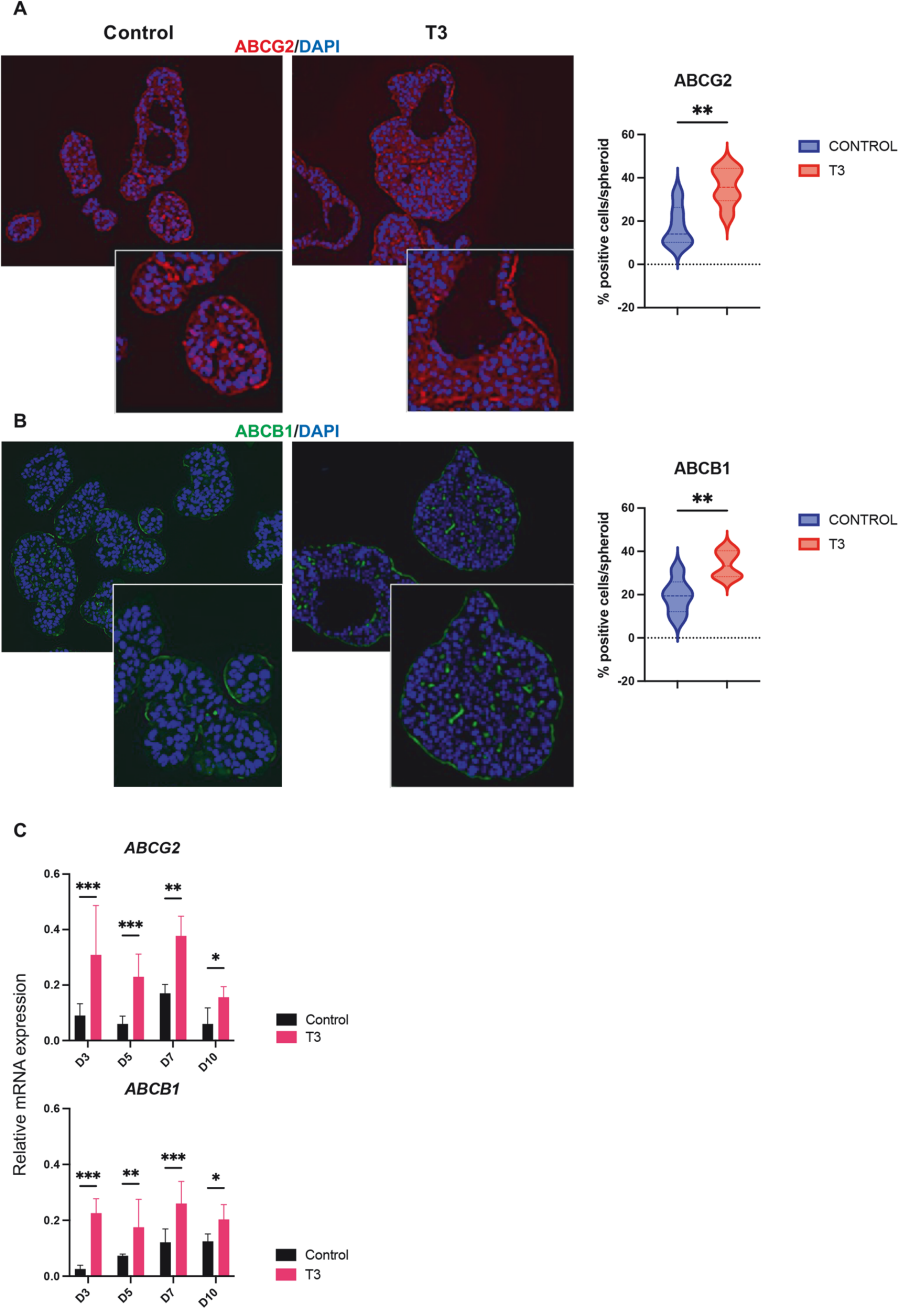
Expression of ABCG2 and ABCB1 is upregulated in T3-treated spheroids. Immunostaining of spheroids at D7 for the ABC transporters ABCG2 (**A**) or ABCB1 (**B**) in different conditions. Left panels: merging between each specific labeling and DAPI (nuclei, blue). Images are representative of at least 3 independent experiments and were taken with a 20× objective under a Zeiss Axio Imager M2 Apotome 2 microscope. Scale bar: low magnification 50 μm, high magnification 25 μm. Right panels: percentage of positive cells per spheroid for each marker in the different conditions. Violin plots show the frequency distribution of the data. Bold dotted lines indicate the median, and light dotted lines indicate the quartiles. *n* = 10 spheroids *per* marker and condition. **P < 0.01 compared to the control condition, by unpaired, two-tailed Student *t*-test. Number of cells scored: ABCG2, Control 343 and T3 1313; ABCB1, Control 414 and T3 1034. **C** Results of RT-qPCR to analyze the mRNA levels of *ABCG2 and ABCB1* in spheroid cultures at different time points in the different conditions. Histograms represent mean ± SD, *N* = 6, after normalization with *PPIB*. *P < 0.05, **P < 0.01, and ***P < 0.001 compared to the control condition by multiple, two-tailed unpaired Student t-test. Results are representative of two independent experiments.

To further investigate features of the spheroids associated with SC/CSC biology, we analyzed the expression of the ABC transporters ABCG2 and ABCB1 [37, 38]. RNA-seq analyses showed that their expression was increased by T3 treatment (Table S3). As expected, ABCG2 and ABCB1 proteins were located at the basal and lateral membranes of the cells. Within the spheroids, ABCG2- and ABCB1-expressing cells were localized on the external surface and in the internal layers surrounding the lumens of spheroids (Fig. 3A, B). The percentage of ABC-expressing cells strongly increased in T3 condition compared with control (Fig. 3A, B). Quantitative analyses by Western blots at D7 (see later) and RT-qPCR at each time point showed increased expression of both proteins and mRNAs in T3-treated spheroids compared to respective controls (Fig. 3C).

Taken together, the presence of T3 during spheroid formation increased the size of spheroids and favored the appearance of bigger lumens, due to the T3-dependent increase of proliferating cells and cells with CSC behavior.

### Effects of altered TRα1 levels on spheroid formation and growth

**Fig. 4:**
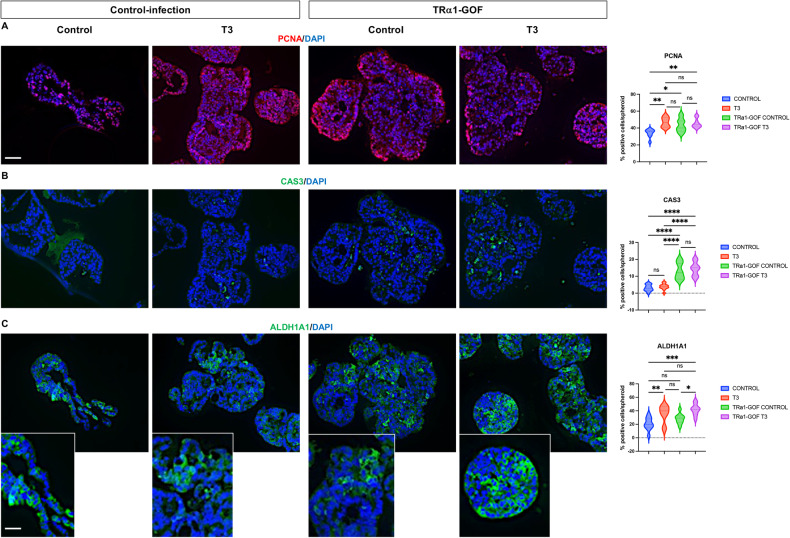
Impact of TRα1 overexpression in spheroids. Immunostaining of spheroids at D7 for markers of: proliferation, PCNA (**A**), cell death, Activated-caspase 3 (CAS3) (**B**), and CSC-like, ALDH1A1 (**C**) in infection-control or TRα1-GOF conditions. Left panels: merging between each specific labeling and DAPI (nuclei, blue). Images are representative of at least 3 independent experiments and were taken with a 20× objective under a Zeiss AxioImager M2 Apotome 2 microscope. Scale bar: low magnification 50 μm, high magnification 25 μm. Right panels: percentages of positive cells per organoid for each marker in the different conditions. Violin plots show the frequency distribution of the data. Bold dotted lines indicate the median, and light dotted lines indicate the quartiles. *n* = 10 spheroids *per* marker and condition. ns: not significant, *P < 0.05, **P < 0.01, ***P < 0.001 and ****P < 0.0001 in the indicated comparisons by 2-way ANOVA. Number of cells scored: PCNA, Control 551, T3 1179, TRα1-GOF Control 802, TRα1-GOF T3 1089; CAS3, Control 773, T3 1194, TRα1-GOF Control 802, TRα1-GOF T3 1064; ALDH1A1, Control 450, T3 725, TRα1-GOF Control 803, TRα1-GOF T3 930.

To investigate the role of TRα1 on spheroid growth and CSC biology, we modulated TRα1 expression in Caco2 cells before generating the spheroids. The parental cell line was transduced to express TRα1 or shRNA for silencing, and successful TRα1 modulation was validated by RT-qPCR (Figs. S1 and S2). First, we investigated the impact of TRα1 gain of function (GOF) and/or of the T3 treatment in the forming step on the spheroids’ growth (Fig. 4; Fig. S1). TRα1 GOF alone stimulated the growth of the spheroids at all time points. T3 in the control-infection group increased the size of spheroids, and the effect was even more dramatic in TRα1 GOF cultures (Fig. S1B, C). TRα1 GOF spheroids had a compacted multilayered structure with small or absent lumens at all time points. However, when T3 was added during the formation of the spheroids, lumens became larger (Fig. S1D). Morphologic changes in control-infected spheroids, with or without T3 treatment, were the same as previously observed in Control/T3 spheroids (Fig. S1D).

Next, we analyzed whether TRα1 GOF could affect proliferation, cell death, and CSCs at D7 by immunolabeling, using the same markers as in the previous experiments. In control-infection spheroids, T3 increased the percentage of PCNA-positive proliferating cells (Fig. 4A). TRα1 GOF strongly increased the rate of cell proliferation compared to control-infection cells (Fig. 4A), while T3 treatment did not further change the percentage of PCNA-positive cells. In relation to apoptosis, the rate of activated-CAS3-positive cells was similar in all conditions (Fig. 4B). ALDH1A1-positive cells were increased in T3 or TRα1 GOF conditions compared to untreated control-infection cells, and further increased in T3-treated TRα1 GOF spheroids (Fig. 4C).

**Fig. 5:**
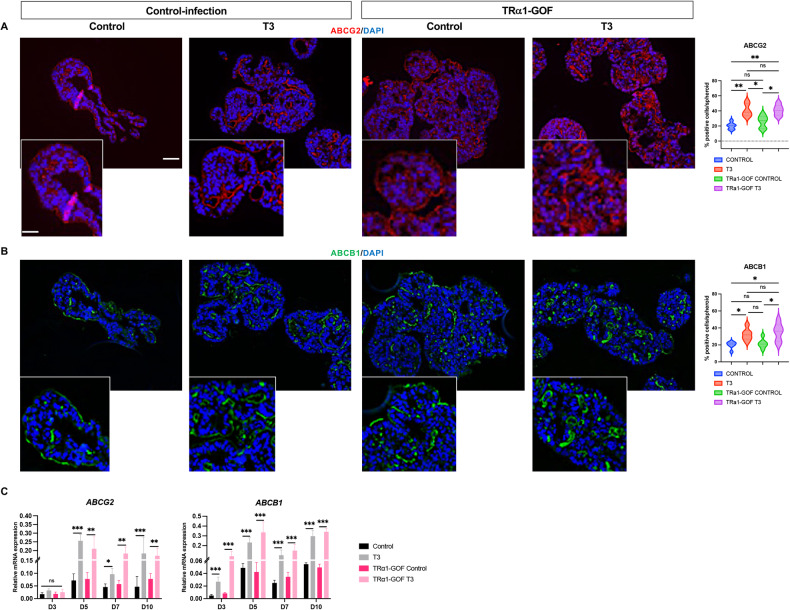
Expression of ABCG2 and ABCB1 in TRα1-GOF spheroids. Immunostaining of spheroids at D7 for the ABC transporters ABCG2 (**A**) or ABCB1 (**B**) in different conditions. Left panels: images show the merging between each specific labeling and DAPI (nuclei, blue) and are representative of at least 3 independent experiments. Images were taken with a 20× objective under a Zeiss AxioImager M2 Apotome 2 microscope. Scale bar: low magnification 50 μm, high magnification 25 μm. Right panels: percentage of positive cells per spheroid for each marker in the different conditions. Violin plots show the frequency distribution of the data. Bold dotted lines indicate the median, and light dotted lines indicate the quartiles. *n* = 10 spheroids *per* marker and condition. ns not significant, *P < 0.05 and ***P < 0.01 compared to the control condition, by 2-way ANOVA. Number of cells scored: ABCG2, Control 444, T3 448, TRα1-GOF Control 563, TRα1-GOF T3 556; ABCB1, Control 416, T3 388, TRα1-GOF Control 512, TRα1-GOF T3 453. **C** RT-qPCR results showing mRNA levels of *ABCG2 and ABCB1* in spheroid cultures at different time points in the different conditions. Histograms represent mean ± SD, *N* = 6, after normalization with *PPIB*. ns: non-significant, *P < 0.05, **P < 0.01, and ***P < 0.001 in the indicated comparisons by 2-way ANOVA. Results are representative of two independent experiments.

We then immunolabeled ABCG2- and ABCB1-expressing cells in different conditions. In both control-infection and TRα1 GOF conditions, T3 treatment increased the percentage of positive cells (Fig. 5A, B). Unlike control-infection cells, they were widely distributed within TRα1 GOF spheroids, not limited to the external layers (Fig. 5A, B). RT-qPCR analyses confirmed that T3 treatment significantly stimulated the expression of *ABCB1* and *ABCG2* mRNA in both the control-infection and TRα1 GOF conditions at all time points (Fig. 5C). When TRα1 was downregulated, spheroid growth was strongly blunted and cells became unresponsive to T3 (Fig. S2). These results corroborated our previous work where we demonstrated, in 2D Caco2 cells, that TRα1 depletion affected cell growth and migration [18].

In conclusion, these results point to the stimulatory functions of TRα1 on the development and growth of the spheroids. Interestingly, the overexpression of TRα1 induced a compact spheroid morphology organized in multilayered cells that could be partially reversed by T3 treatment. This phenotype was correlated with increased expression of ALDH1A1 and of ABC transporters.

### T3 modulates the response of spheroids to chemotherapy

**Fig. 6:**
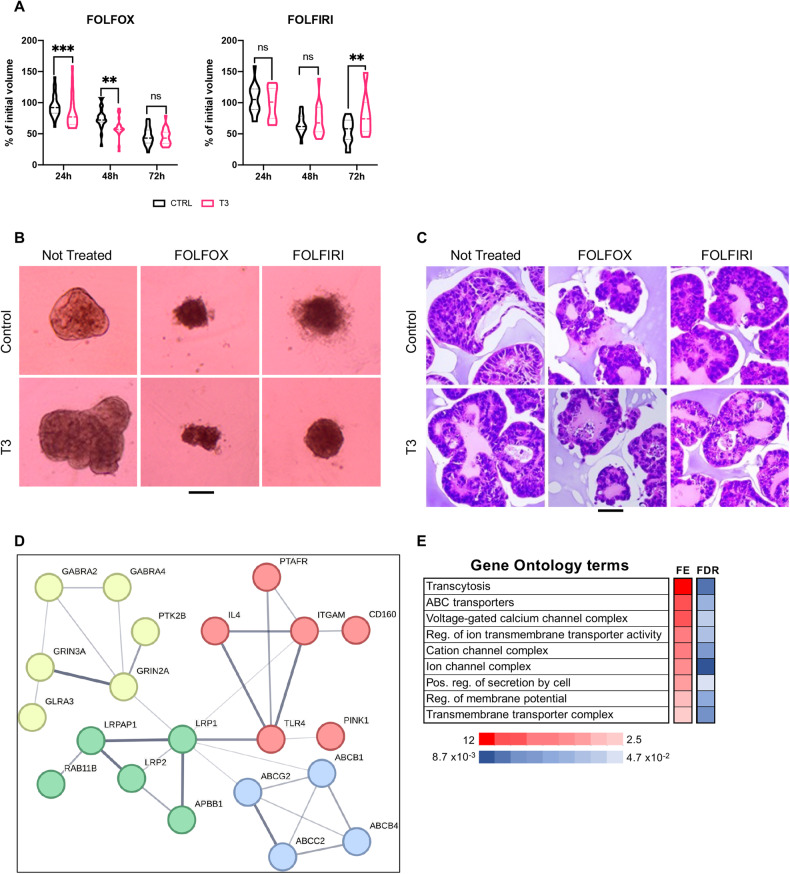
Effects of anticancer regimens in control and T3 spheroids. **A** Changes in volume of spheroids shown as the percentage of initial volumes at the indicated time points after drug treatments. Violin plots show the frequency distribution of the data. Bold dotted lines indicate the median and light dotted lines indicate the quartiles, *n* = 12. ns non-significant, **P < 0.01 and ***P < 0.001 compared to the respective control conditions by multiple unpaired, two-tailed Student *t*-test. Results are representative of two independent experiments. **B** Morphologic features of the spheroid cultures in the different conditions. Images were taken under a Zeiss AxioVert microscope with a 4X objective. Scale bar: 200 μm. **C** H&E staining of paraffin sections. Representative images of the control and T3 spheroids untreated or after 72 h of FOLFOX or FOLFIRI treatments. Images were taken using a Zeiss AxioImager with a 20X objective. Scale bar: 50 μm. Comparative transcription profiles by RNA-seq showing network analysis of unique upregulated genes in T3-FOLFIRI condition (**D**) and GO analysis (**E**). Note the presence of several ion channels and ABC drug transporters.

Because we observed increased expression of two drug efflux pumps in T3-treated spheroids, we investigated whether T3 treatment might influence response to commonly used chemotherapy in CRCs. We first studied the impact of T3, alone or combined with FOLFOX or FOLFIRI, on spheroid growth. On D3, 24 h after harvesting, spheroids were maintained in a control condition or treated with FOLFOX or FOLFIRI for 72 h. Every 24 h, we measured changes in spheroid volume compared with the initial volume on D3. FOLFOX-treated spheroids had significantly decreased volume in both control and T3 conditions at 48 h and 72 h (Fig. 6A, left panel). After 72 h of treatment with FOLFOX, the morphology of spheroids appeared similarly affected in both control and T3 conditions and detached dead cells were present in the plates (Fig. 6B). FOLFIRI-treated spheroids in the control condition had decreased volume at both 48 and 72 h. T3 plus FOLFIRI treatment resulted in a minor and non-significant change in cell volume at 24 and 48 h, but volumes returned to control levels at 72 h (Fig. 6A, right panel). These observations were confirmed at the morphologic level (Fig. 6B).

To better appreciate the histology and features of spheroids in the different conditions, after 72 h of treatment with FOLFIRI or FOLFOX, we performed H&E staining and IF analyses on paraffin sections and molecular analyses. Control and T3-treated spheroids, in the absence of chemotherapy, had similar morphology as shown before, and T3 induced a slightly increase in size (Fig. 6C). FOLFOX-treated spheroids with or without T3 were smaller and had amorphous shapes (Fig. 6C). FOLFIRI spheroids without T3 treatment showed a multilayered morphology with small lumens. In contrast, FOLFIRI spheroids with T3 treatment maintained an almost intact morphology of mixed multilayered and monolayered zones with the presence of internal lumens (Fig. 6C).

To define the molecular mechanisms responsible for these results, we conducted RNA-seq analyses (Tables S4–S6). Figure S3A, B summarize the criteria applied to select the differentially up- or downregulated genes for FOLFIRI (Fig. S3A or B, left panels) or FOLFOX (Fig. S3A or B, right panels). Briefly, in FOLFIRI or FOLFOX conditions, up- or downregulated genes in control and T3-treated spheroids were compared with genes expressed in control spheroids (without T3 and without chemotherapy) to obtain shared and specific gene sets. Then, we compared the T3-modulated genes without FOLFIRI or FOLFOX with the control condition to define genes regulated by T3, independent of chemotherapies. Results of Gene Ontology and Metascape analyses on each gene set are shown in Tables S4-S6. Since FOLFIRI plus T3 resulted in a resistant phenotype, we analyzed that condition in more detail.

We compared up- and downregulated gene sets in FOLFIRI and FOLFOX and retained only those uniquely present in the FOLFIRI condition, named unique T3-FOLFIRI Up or T3-FOLFIRI Down sets (Fig. S3A, B; Table S7). Unique sets of genes in the T3-FOLFIRI Up set included genes involved in controlling cell metabolism and energy production, transcytosis/endocytosis/drug efflux, and cell junctions (Fig. 6D, E; Table S7). In particular, the gene network depicted in Fig. 6D shows multiple genes encoding for proteins involved in cell detoxification, such as the ABC transporters and membrane-associated ion channels. The uniquely downregulated genes in T3-FOLFIRI are associated with stress response, cell cycle regulation, cell division, and DNA metabolism (Fig. S4; Table S7). This result is congruent with the stress-induced and anti-metabolic actions of 5-FU and irinotecan [22, 39].

### T3 promotes survival and maintains proliferative capacity of FOLFIRI-treated spheroids

**Fig. 7:**
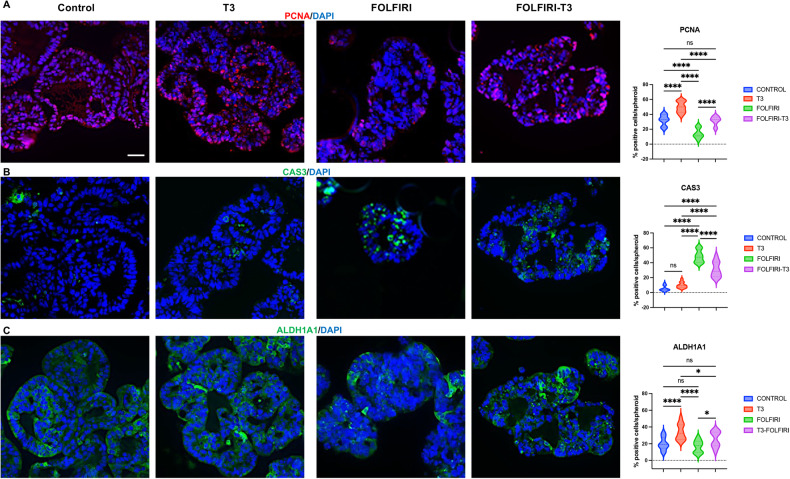
Effects of T3 plus FOLFIRI treatments in spheroids. Immunostaining of spheroids in the control and T3 groups and then after 72 h with or without FOLFIRI. Markers of: proliferation, PCNA (**A**), cell death, Activated-caspase 3 (CAS3) (**B**), and CSC-like ALDH1A1 (**C**) were analyzed. Left panels: merging between each specific labeling and DAPI (nuclei, blue) representative of at least 3 independent experiments. Images were taken with a 20× objective under a Zeiss AxioImager M2 Apotome 2 microscope. Scale bar: 50 μm. Right panels: percentage of positive cells per organoid for each marker in the different conditions. Violin plots show the frequency distribution of the data. Bold dotted lines indicate the median and light dotted lines indicate the quartiles. *n* = 10 spheroids *per* marker and condition. ns: not significant, *P < 0.05 and ****P < 0.0001 in the indicated comparisons by 2-way ANOVA. Number of cells scored: PCNA, Control 741, T3 581, FOLFIRI 384, FOLFIRI T3 423; CAS3, Control 721, T3 576, FOLFIRI 379, FOLFIRI T3 423; ALDH1A1, Control 617, T3 532, FOLFIRI 467, FOLFIRI T3 390.

The morphologic and molecular features of the spheroids treated with T3 plus FOLFIRI prompted us to analyze their characteristics linked to cell proliferation, cell death, and CSCs in more detail. In non-treated conditions, T3 induced the rate of proliferating cells but did not affect apoptosis compared with controls (Fig. 7A, B). In the FOLFIRI condition, proliferating PCNA-expressing cells significantly decreased and activated-CAS3 apoptotic cells increased (Fig. 7A, B). However, with T3 treatment in the FOLFIRI condition, the percentage of proliferating cells was similar to untreated controls and there were fewer apoptotic cells than in the FOLFIRI condition (Fig. 7A, B). The use of another marker of cell proliferation, Cyclin D1, essentially confirmed the results obtained with PCNA (Fig. S5). FOLFIRI treatment did not affect the number of ALDH1A1-positive, possibly CSC-like cells, while T3 treatment had a slight stimulatory and statistically significant effect compared with the FOLFIRI alone condition (Fig. 7C).

**Fig. 8:**
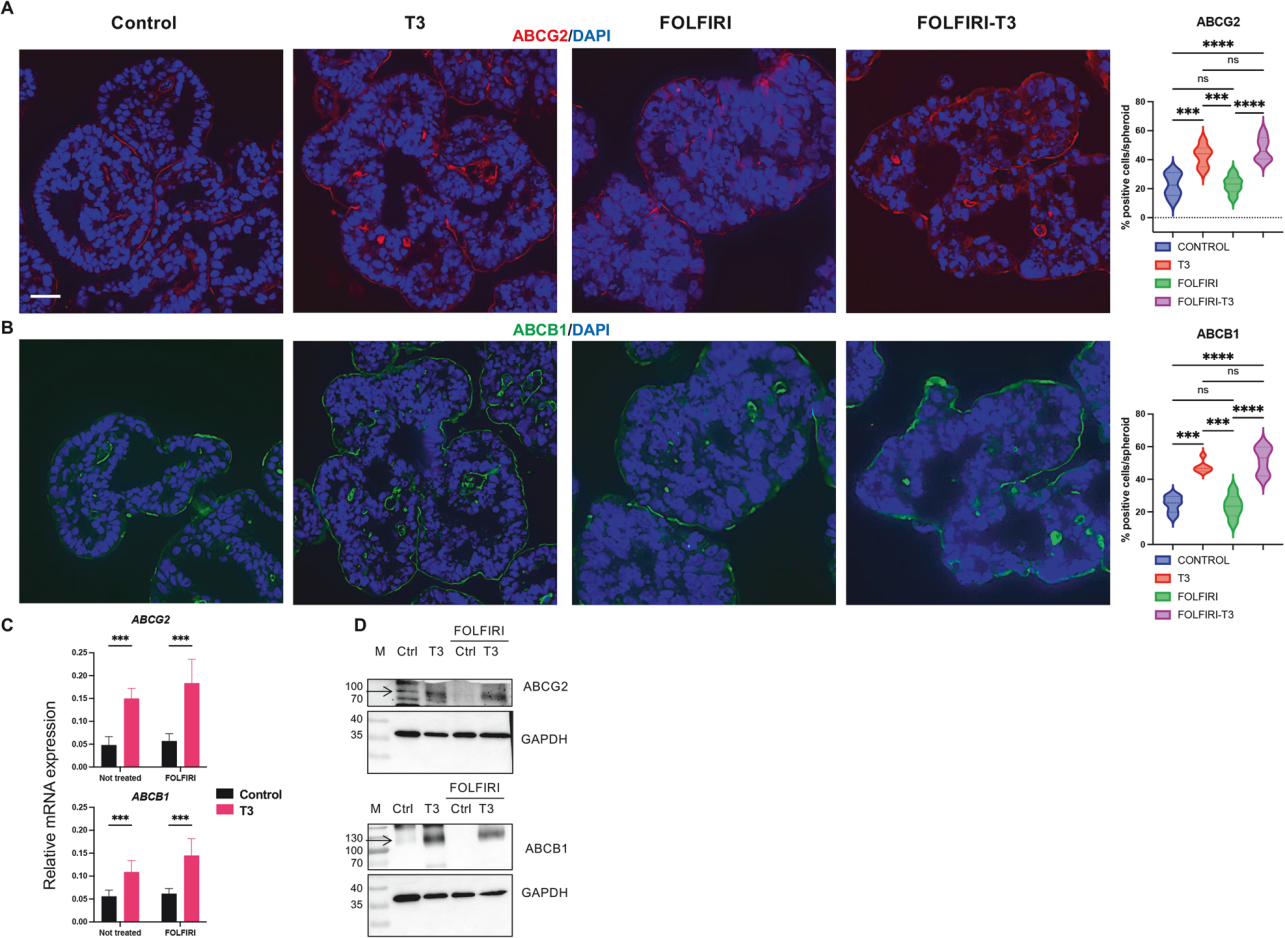
T3 stimulates the expression of ABCG2 and ABCB1 in FOLFIRI-treated condition. **A**, **B** Immunostaining of spheroids in the control and T3 groups and then after 72 h with or without FOLFIRI. ABC transporters ABCG2 (**A**) or ABCB1 (**B**) were analyzed in different conditions. Left panels: images show the merging between each specific labeling and DAPI (nuclei, blue) and are representative of at least 3 independent experiments. Images were taken with a 20X objective under a Zeiss Axio Imager M2 Apotome 2 microscope. Scale bar: 50 μm. Right panels: percentage of positive cells per spheroid for each marker in the different conditions. Violin plots show the frequency distribution of the data. Bold dotted lines indicate the median, and light dotted lines indicate the quartiles. n = 10 spheroids *per* marker and condition. ns not significant, ***P < 0.001 and ****P < 0.0001 compared to the control condition, by 2-way ANOVA. Number of cells scored: ABCG2, Control 403, T3 336, FOLFIRI 286, FOLFIRI T3 251; ABCB1, Control 458, T3 340, FOLFIRI 301, FOLFIRI T3 273. **C** Results of RT-qPCR experiments showing expression levels of *ABCG2 and ABCB1* mRNAs in different conditions. Histograms represent mean ± SD, *N* = 6, after normalization with *PPIB*. ***P < 0.001 in the indicated comparisons by 2-way ANOVA. Data are representative of two independent experiments. **D** Western blot analysis of ABCG2 and ABCB1 in spheroids maintained in the different conditions. GAPDH was used as the loading control. Data are representative of two independent experiments. Arrows in ABCG2 and ABCB1 blots indicate the specific bands.

To complete the study of CSCs, we immunolabeled ABCG2- and ABCB1-expressing cells in the different experimental conditions. As for the previous analysis, T3 significantly stimulated the number of ABC transporter-positive cells (Fig. 8A, B), but FOLFIRI had no clear-cut effect on number or distribution of those cells compared with the control condition. T3 treatment in the FOLFIRI condition increased, however, the percentage of ABC transporter-positive cells (Fig. 8A, B). RT-qPCR analyses confirmed T3 stimulation of *ABCB1* and *ABCG2* mRNA in both control and FOLFIRI conditions (Fig. 8C). A similar result was obtained in spheroids with altered TRα1 levels (Fig. S6). The stimulatory effects of T3 on both ABCG2 and ABCB1 protein expression, in control or FOLFIRI conditions, were confirmed by Western blot analyses (Fig. 8D).

Altogether, these studies showed that T3 pre-treatment during spheroid formation primes the cells toward a resistant phenotype and is linked to increased expression of ABC transporters that are responsible for stimulated detoxification. Importantly, FOLFIRI and/or T3 treatment(s) affected both TRα1 expression and that of the UGT1A enzyme, which is involved in the inactivation and detoxification of the irinotecan present in FOLFIRI combination [40]. On one hand, FOLFIRI (but not FOLFOX) induced the expression of TRα1 independently from T3 (Fig. S7A–C). On the other hand, T3 in the FOLFIRI condition counteracted the decreased UGT1A levels induced by FOLFIRI alone (Fig. S8).

Taken together, we found that stimulating TRα1 action through T3 reduces the anti-metabolic effects of FOLFIRI treatment by acting on protein transporters, ion channels, and cellular enzymes.

## Discussion

Our studies aimed at understanding the effects of the thyroid hormones on colon CSCs, including their impact on the response to chemotherapy. We demonstrated that the thyroid hormone T3 and the nuclear hormone receptor TRα1 influence a) the development and growth of spheroids derived from the Caco2 colon adenocarcinoma cell line, and b) their response to FOLFIRI, a chemotherapeutic regimen used for treating CRC patients. These findings provide the first evidence that analyzing thyroid hormone status of patients could optimize chemotherapeutic treatments for CRC.

We observed that the presence of T3 during the stage of spheroids formation primes them in several ways, modifying their morphology and molecular features several days after the exposure. Our previous studies showed a “thyroid shock” in mouse intestinal organoids treated with T3 [41], also responsible for high metabolic reprogramming and the modifications of SC markers. Similarly, in this study, the T3-treated spheroids were larger and displayed differential expression of some CSC markers during the days in culture. The most striking observation in this study is that T3-treated spheroids increased the expression of two ABC transporters, ABCB1 and ABCG2, well-known drug efflux pumps involved in drug resistance [25, 42]. The overexpression of TRα1 showed similar results and underlined that the action of TRα1 to induce the ABC transporters depends on T3. Finally, although the direct transcriptional regulation of *ABCB1* by T3/TRα1 had been reported [28, 37, 38], no information was available for *ABCG2*. An *in-silico* analysis of putative nuclear hormone receptor binding sites (Nubiscan) suggested a thyroid hormone binding site within 8 Kb of the *ABCG2* promoter, potentially explaining the molecular basis of this control (our unpublished observation). It is worth noting that ALDH1A1 and ABCG2 have been reported as markers of CSC-like cells in several settings [43–45]. Thus, similar to our results in normal intestine [41] these new data strongly suggest that T3 and TRα1 also impact CSC-like cells. However, we cannot exclude an effect on other cancer cell types, given that ABCB1 expression is also associated with the differentiated intestinal epithelial cells [46].

Recent studies have underlined the activation of an embryonic program, named diapause, after FOLFIRI treatment in vivo and in colon cancer spheroids and tumoroids [47]. In embryos, this program is activated under growth stress and/or nutrient deprivation. The cells undergo quiescence, and the embryo stops growing, but the state is reversed when environmental conditions become favorable. Chemotherapies, particularly FOLFIRI, induce a similar program, and drug-persistent cells enter a diapause-like (DTP-like) status. These cells are not chemo-resistant, since they can successfully re-enter a DTP-like state after treatment [47, 48]. We were intrigued by this program induced by FOLFIRI and compared the genes of the DTP-like signature defined by Rehman et al. [47] to our unique FOLFIRI gene sets (up or down). However, we found only a marginal overlap, essentially limited to the ABC transporters (our unpublished observations). We speculate that T3, a metabolism-stimulating hormone [49, 50], maintains the cells in a more active status than the DTP-like induced by FOLFIRI alone. This assumption is reinforced by the maintenance of basal proliferation activity, as underlined by analyzing the PCNA- or Cyclin D1-positive cells in T3-FOLFIRI spheroids relative to FOLFIRI alone. However, when compared to control spheroids, the overall metabolic status of T3-FOLFIRI spheroids is affected, as shown in Fig. S4 and in Table S7. It is worth underlining, however, that a similar analysis using Ki67 antibodies gave a less clear result (our unpublished observation). This can be due to a larger expression profile of Ki67 during cell cycle or to its additional roles in cells, beyond its involvement in cell cycle [51]. Finally, our study focused on protein-coding RNAs, but we found several differentially regulated long-non coding (Lnc)-RNAs in our data-sets (Tables S8 and S9). This result highlights the potential existence of additional mechanisms responsible for T3/TRα1-mediated drug resistance.

We selected Caco2-derived spheroids for our studies due to the capacity of Caco2 cells to integrate and respond to T3 [18] and the presence of different cell states, including proliferating, differentiated, and CSC-like cells [30]. Since some heterogeneity was present in these spheroids, mimicking colon cancer, we also tested whether T3 plus FOLFIRI could modulate the survival of cells from patient-derived tumoroids. For this aim, we performed the analysis on tumoroids generated from a poorly differentiated tumor [52], a mesenchymal tumor type. In this case, however, FOLFIRI-induced cell death could not be rescued by T3 (our unpublished observations). More in-depth analyses of patient-derived tumoroids generated from various tumor differentiation statuses will help define which tumor types can respond to T3 and bypass the FOLFIRI treatment. Such an investigation, coupled with the analysis of TRα1 expression level in the tumor, may improve CRC diagnoses.

In conclusion, our study showed for the first time that T3 and TRα1 confer a phenotypic survival advantage for colon CSC-like and other tumor cells. However, this advantage depends on the drug combination administered, since it occurs only in the presence of FOLFIRI but not of FOLFOX.

## Materials and methods

### Cell cultures

The human adenocarcinoma cell line Caco2 (from ATCC) was cultured in DMEM Glutamax (4.5 g/l D-Glucose with pyruvate) medium (Gibco) supplemented with 10% heat-inactivated fetal bovine serum (FBS, Gibco) and 1% penicillin/streptomycin (P/S) (Gibco) at 37 °C in a humidified atmosphere containing 5% CO_2_.

### Spheroid cultures

Spheroids were generated, cultured, and harvested as previously reported [30]. Briefly, 600 Caco2 cells per spheroid were seeded in an Aggrewell plate (StemCell Technologies) and cultured for 48 h before harvesting. Then the spheroids were placed in agarose-coated plates for successive analyses. To evaluate T3 response, during the 48 h of spheroid formation, 10^−7 ^M T3 (Sigma) was added to the spheroid culture medium. After harvesting, all spheroids were cultured in the same spheroid medium (DMEM supplemented with 2.5% Matrigel™ (Corning), 10% FBS, and 1% P/S).

The growth of spheroids was monitored under a Zeiss AxioVert inverted microscope and images were analyzed using ImageJ software. Three representative diameters of each structure were measured, and the sphere volume formula was used to obtain the estimated volume of the spheroids, as previously reported [30].

### Treatment of spheroids with chemotherapeutic drugs

Spheroids were harvested after 48 h with or without T3 (10^−7 ^M) and plated in agarose-coated plates. One day after harvest from Aggrewell (D3), they were treated with FOLFIRI (5-FU, 50 µg/mL; irinotecan, 100 µg/mL; leucovorin, 25 µg/mL) or FOLFOX (5-FU, 50 µg/mL; oxaliplatin, 10 µg/mL; leucovorin, 25 µg/mL), two chemotherapeutic regimens routinely used to treat CRC patients [20, 53]. Untreated spheroids were maintained as controls.

### Modulation of TRα1 expression

These studies used the human colon adenocarcinoma cell line Caco2. For the loss-of-function experiments, Mission-shRNA (derived from pLKO.1-puro, Sigma) lentiviral vectors were used. The Sh sequences targeting TRα1 are listed in Table S1. For overexpression experiments, the TRα1 cDNA was inserted in pLKO.1-puro vector (Sigma). The cells were cultured with the lentiviral particles for 24 h and maintained for an additional 24 h before being used to generate the corresponding spheroids. The lentiviral particles were produced by the AniRA facility (SFR Biosciences, Lyon, France).

### Immunolabeling, histologic staining, and cell scoring

For histologic and immunolabeling analyses, the spheroids were collected at different time points and fixed in 2% PFA before inclusion in paraffin for sectioning and hematoxylin and eosin (H&E) staining or immunolabeling. Paraffin inclusions and sections were performed by the Anipath Recherche facility (CRCL/CLB, Lyon, France). 5-μm thick sections were used for indirect immunostaining. Briefly, the sections were deparaffinized in methylcyclohexane, hydrated in ethanol (100%, 90%, and 75%), and washed with PBS. *For immunofluorescence*, the slides were subsequently subjected to antigen retrieval using a Tinto Retriever Pressure Cooker (Bio SB) in 0.01 M citrate buffer, pH 6, and incubated for one hour at room temperature with blocking buffer (10% normal goat serum, 1% BSA, and 0.02% Triton X-100 in PBS). The slides were then incubated with primary antibodies overnight at 4 °C, followed by incubation with fluorescent secondary antibodies (Alexa Fluor, Life Technologies). Finally, slides were mounted using a fluoro-gel mounting medium with DAPI (Interchim, FP-DT094B). Fluorescence microscopy and imaging were performed on a Zeiss AxioImager Apotome M2. *For immunohistochemistry*, after deparaffinization and dehydration, tissue sections were heated for 50 min at 97 °C in 10 mM citrate buffer, pH 6.0. To block endogenous peroxidases, tissue sections were incubated in 5% hydrogen peroxide solution. IHC was performed on an automated immunostainer (Ventana Discovery XT; Roche) using an Omnimap DAB Kit according to the manufacturer’s instructions and sections were incubated with the primary antibodies. The secondary anti-rabbit-HRP antibody was applied to the sections, and staining was visualized with DAB solution with 3,3′-diaminobenzidine as a chromogenic substrate. Finally, the sections were counterstained with Gill’s hematoxylin and then scanned with a Panoramic Scan II (3D Histech, Budapest, Hungary) at 20X. The antibodies used are listed in Table S2.

For each labeling, cell scoring has been performed manually in blinded manner by using Image J software on a minimum of 10 spheroids per condition. The number of total cells counted is reported in each figure legend.

### RNA extraction and RT-qPCR analyses

Total RNA was extracted using the Nucleospin RNA XS Kit (Macherey-Nagel). DNase digestion was performed in all preparations to avoid contamination by gDNA. We performed reverse transcription with iScript reverse transcriptase (Bio-Rad) on 500 ng of total RNA, according to the manufacturer’s instructions. For qPCR approaches, the SYBR qPCR Premix Ex Taq II (Tli RnaseH Plus) (Takara) was used in a CFX connect apparatus (Biorad). Specific mRNA expression was quantified using the ΔCt/ΔΔCt method, and values were normalized to *PPIB* levels. Primers are listed in Table S1.

### Protein analysis by western blot

Protein samples from spheroids were prepared with RIPA buffer as previously described [19], separated by SDS–PAGE (50 μg per lane), and transferred to 0.2 μm PVDF membranes (Bio-Rad). Blots were incubated in a blocking buffer (PBS-Tween supplemented with 5% non-fat milk) and then with primary antibodies. This step was followed by incubation with HRP-conjugated secondary antibodies (Promega). Signals were analyzed using an enzymatic Clarity Substrate Detection Kit and Clarity Max ECL (Bio-Rad), according to the manufacturer’s protocol. For image detection, we used a Pixie imaging system (Gene-sys). The antibodies are listed in Table S2.

### Transcriptome analyses

#### Sample preparation for sequencing

(1) To study the effect of T3 during spheroids formation, we generated spheroids in Aggrewell plates [30] treated or not with 10^−7 ^M T3 for 24 h before recovery (2). To study the combined effects of T3 and chemotherapies, spheroids were generated with or without T3 for 48 h before harvesting. They were then transferred to agarose-coated plates, and FOLFOX or FOLFIRI was added 24 h after harvesting for a total of 72 h. Spheroids in different conditions were recovered as dry pellets and used for RNA extraction and sequencing (Active Motif RNA-seq service; www.activemotif.com). Briefly, (1) isolation of total RNA, (2) analysis of RNA quality/integrity, (3) library generation, and (4) sequencing were performed using the Illumina platform, as we described previously [32]. The data were successively processed to define differentially expressed genes. ENA (https://www.ebi.ac.uk/ena/browser/home) submission number: PRJEB71636.

#### Additional bioinformatics analyses

##### Differential gene expression analyses

Using FASTq files from RNA-seq assays, we quantified gene expression using Kallisto (v0.43.1) [54] and the R package tximport (v1.0.3) [55]. GENCODE (v37, https://www.gencodegenes.org) was used as a reference for the human transcriptome. Differential gene expression analyses between groups were performed with DESeq2 v3.6.2 [56]. Genes with |log2FoldChange| ≥ 0.5 and adjusted p-value (FDR) < 0.05 were considered as differentially expressed.

##### Gene ontology and pathway analysis

Genes that were determined to be differentially expressed in the drug treatment (FOLFIRI, FOLFOX, or T3) analyses were evaluated with ShinyGO and Metascape to identify enriched biological processes and pathways and drug treatments producing similar results [57, 58].

### Statistical analyses

Statistical analyses of data represented in graphs were conducted using GraphPad Prism software (version 10; GraphPad Software Inc., San Diego, CA, USA). Tests were performed to analyze the statistical significance between groups, with significance established as a *p*-value < 0.05. The description of the specific test applied is added in each figure legend.

### Supplementary information

Supplementary Informations Merged

Original Data WBs

Table S1 and S2

Table S3

Table S4

Table S5

Table S6

Table S7

Table S8

Table S9

## Data Availability

Data are available upon request to the corresponding author.
