# Supplementary material for: Impact of the thyroid hormone T3 and its nuclear receptor TRα1 on colon cancer stem cell phenotypes and response to chemotherapies
Source: Cell Death Dis. 2024 May 1;15(5):306. doi: 10.1038/s41419-024-06690-x (PMC11063186; doi:10.1038/s41419-024-06690-x)

Supplementary Figure Legends

Figure S1: Effects of TRα1 overexpression on spheroid formation and growth. A) RTqPCR analyses showing increased TRα1 mRNA levels in TRα1-infected cells compared with control-infection. Histograms represent mean ± SD, N = 6, after normalization with *PPIB*. *: $P<0.05$ compared to controls by unpaired, two-tailed Student t-test. Results are representative of three independent experiments. **B)** Representative images of TRα1-GOF spheroid cultures at different time points in different conditions. Images were taken under a Zeiss AxioVert microscope with a 4X objective. Scale bar: 200 µm. **C)** Estimated volume of TRα1 GOF spheroids in different conditions and at different time points. Violin plots show the frequency distribution of the data. Bold dotted lines indicate the median and light dotted lines indicate the quartiles, N = 16. ns: not significant, *: $P<0.05$ and **: $P<0.01$ compared to the indicated condition by multiple unpaired, two-tailed Student t-test. Results are representative of two independent experiments. **D)** H&E staining of paraffin sections. Data shown are representative images of spheroids at the indicated time points after harvesting. Scale bar: 50 µm.

Figure S2: Effects of TRα1 downregulation on spheroid growth. A) Results of RTqPCR analyses showing decreased TRα1 mRNA levels. We used two different Sh-RNA sequences targeting TRα1 in infected cells compared with Scr-Sh control-infection cells. Histograms represent mean ± SD, N = 6, after normalization with *PPIB*. ***: $P<0.001$ and ****: $P<0.0001$ in the indicated comparisons by one-way ANOVA. Results are representative of three independent experiments. **B)** Representative images of spheroids in different conditions at the time points indicated. Images were

taken under a Zeiss AxioVert microscope with a 4X objective. Scale bar: 200 µm. **C)** Estimated volumes of spheroids in the different conditions, as indicated. Violin plots show the frequency distribution of the data. Bold dotted lines indicate the median and light dotted lines indicate the quartiles, N = 12. ns: not significant and **: $P<0.01$ compared to the indicated condition by 2-way ANOVA. Results are representative of two independent experiments. **D)** H&E staining of paraffin sections. Representative images of spheroids at the indicated time points after harvesting are shown. Scale bar: 100 µm.

Figure S3: Selection process of unique gene sets regulated by T3 plus FOLFIRI treatment in spheroids. A, B) Venn diagrams show the unique or overlaps between the up-regulated **(A)** or down-regulated **(B)** genes in the different experimental conditions. For further network and GO analyses we retained only the "Unique T3-FOLFIRI Up" or "Unique T3-FOLFIRI Down" gene sets.

Figure S4: Complementary transcription profile analyses by RNA-seq. **(A)** Network analysis of unique downregulated-regulated genes in T3-FOLFIRI condition and **(B)** GO analysis.

Figure S5: Complementary analysis of cell proliferation in spheroids treated with T3 plus FOLFIRI. Immunostaining for Cyclin D1 of spheroids in the control and T3 groups and then after 72 hr with or without FOLFIRI. Panels show Cyclin D1 labeling (dark brown) and nuclei (light blue) counter-staining. Scale bar: 50 µm. Low panel: percentage of positive cells per spheroid in the different conditions. Violin plots show the frequency distribution of the data. Bold dotted lines indicate the median and

light dotted lines indicate the quartiles, n = 10 spheroids *per* condition. ns: not significant, **: *P*<0.01 and ****: P< 0.0001 in the indicated comparisons by 2-way ANOVA. Number of cells scored: Control 481, T3 427, FOLFIRI 311, FOLFIRI T3 420.

Figure S6: Effects of FOLFIRI on the expression of *ABCG2* and *ABCB1* mRNAs in TRα1-GOF spheroids. Expression levels of the ABC transporters *ABCG2 and ABCB1* mRNAs in different conditions, as analyzed by RT-qPCR. Histograms represent mean ± SD, N = 6, after normalization with *PPIB*. ns: not significant, *: P<0.05, **: *P*<0.01 and ***: *P*<0.001 in the indicated comparisons by 2-way ANOVA. Results are representative of three independent experiments.

Figure S7: Treatment with FOLFIRI specifically increases the expression levels of TRα1. A, B) *TRα1* mRNA levels in the different conditions under FOLFIRI (A) or FOLFOX (B) regimens, as analyzed by RT-qPCR. Histograms represent mean ± SD, N = 12, after normalization with *PPIB*. ns: not significant and **: *P*<0.01 in the indicated comparisons by 2-way ANOVA. Results are representative of two independent experiments. **C, D)** Western blot analysis of TRα1 in spheroids maintained in the different conditions under FOLFIRI **(C)** or FOLFOX **(D)** regimens. Actin was used as the loading control. Images are representative of two independent experiments. The arrow in TRα1 blots indicates the specific band. The numerical values under TRα1 blots represent the normalized quantification of TRα1 on Actin in each lane by densitometry analysis (Image J gel analysis tool). Ctrl: Control, FI: FOLFIRI, T3-FI: T3 plus FOLFIRI, FX: FOLFOX, T3-FX: T3 plus FOLFOX.

Figure S8: T3 plus FOLFIRI treatment of spheroids rescues protein levels of UGT1A enzyme. Western blot analysis of UGT1A in spheroids maintained in the different conditions. Actin was used as the loading control. Images are representative of two independent experiments. Ctrl: Control, F: FOLFIRI, T3-F: T3 plus FOLFIRI.

Figure S9: Original images of the WB illustrated in Figure 8. The blots in A and B were incubated first with anti-ABCG2 or ABCB1 antibodies (upper panels), then stripped and incubated with anti-GAPDH antibodies. For the ABCG2 blot (A), the composite merged image (color and luminescence) resulted in very faint bands, so in Figure 8 we chose to show only the chemoluminescent signal and more specifically the panel ABCG2bis. It was obtained by eliminating the upper part of the blot during the exposure time, resulting in more evident bands at around 72 kDa (black arrow), corresponding to ABCG2. Black dotted rectangles in the left panels indicate the part of the blot illustrated in Figure 8. MW: molecular weight; C: Control, T: T3, F: FOLFIRI, T-F: T3 plus FOLFIRI.

Figure S10: Original images of the WB illustrated in Figure S7. (A, B) The blots were incubated first with anti-TRα1 antibodies as indicated, then stripped and incubated with anti-actin antibodies. Panel A illustrates an experiment conducted with the FOLFIRI regimen, and panel B with the FOLFOX regimen. Black dotted rectangles in each image delimitate the part of the blot illustrated in Figure S7. MW: molecular weight; Ctrl: Control, FI: FOLFIRI, T3-FI: T3 plus FOLFIRI, FX: FOLFOX, T3-FX: T3 plus FOLFOX.

Figure S11: Original images of the WB illustrated in Figure S8. The blot was incubated first with anti-UGTA1 antibodies (upper panel), then stripped and incubated with anti-actin antibodies. Black dotted rectangles in each image delimitate the part of the blot illustrated in Figure S8. MW: molecular weight, Ctrl: Control, F: FOLFIRI, T3-F: T3 plus FOLFIRI.

Supplementary Table Legends

Table S1: Primers used for RT-qPCR and sequence of the Sh used.

Table S2: Antibodies used for immunolabeling and western blot.

Table S3: RNA-seq analyses of T3 treated spheroids during their formation.

Table S4: RNA-seq analyses of T3 treated spheroids without chemotherapies.

Table S5: RNA-seq analyses in spheroids treated with T3 plus FOLFIRI.

Table S6: RNA-seq analyses in spheroids treated with T3 plus FOLFOX.

Table S7: Unique gene sets in spheroids treated with T3 plus FOLFIRI.

Table S8: RNA-seq analyses of Lnc-RNAs in the different conditions.

Table S9: FOLFIRI vs FOLFOX analyses of Lnc-RNAs in the different conditions.

Giolito et al., Figure S1

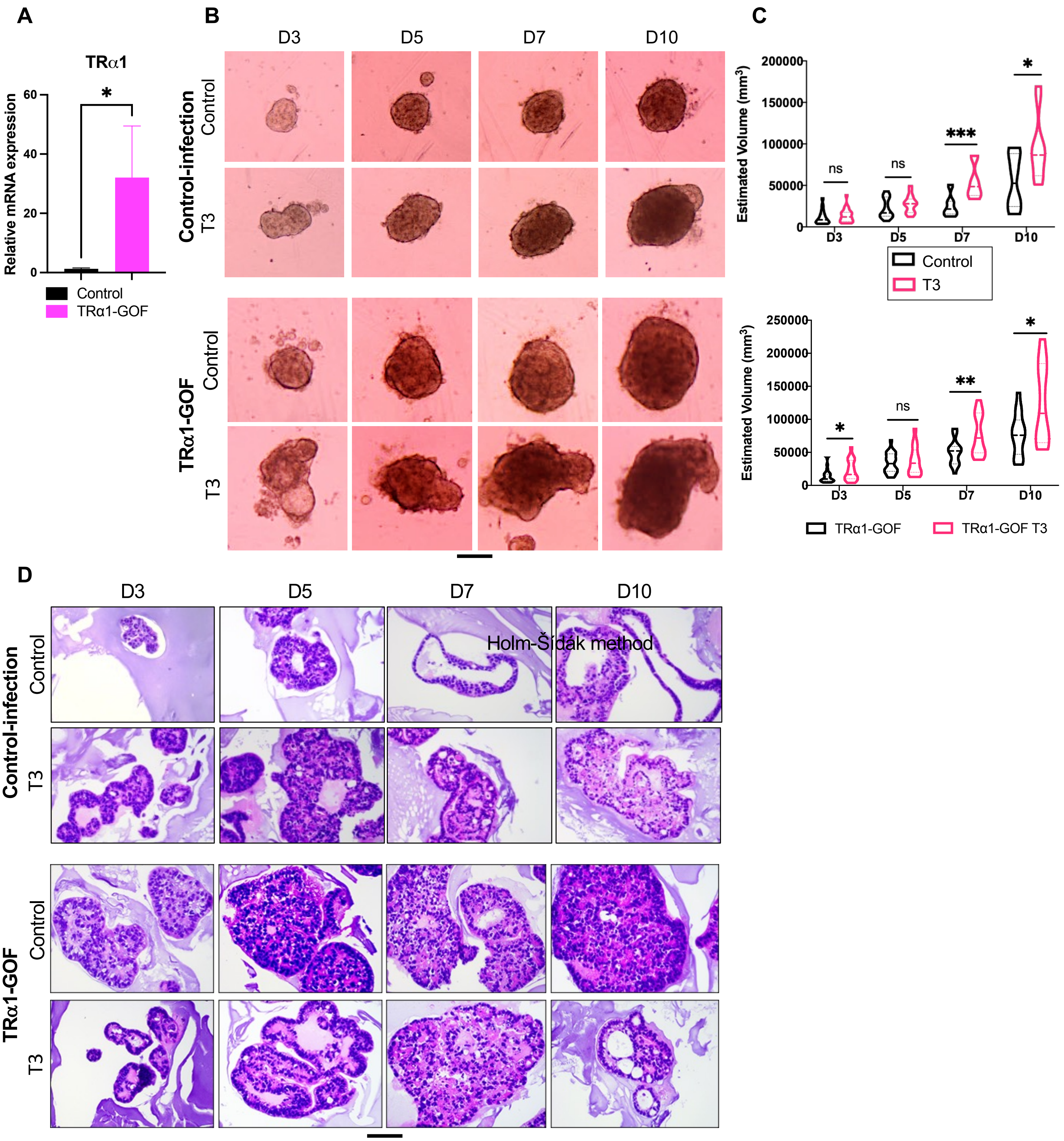

Giolito et al., Figure S2

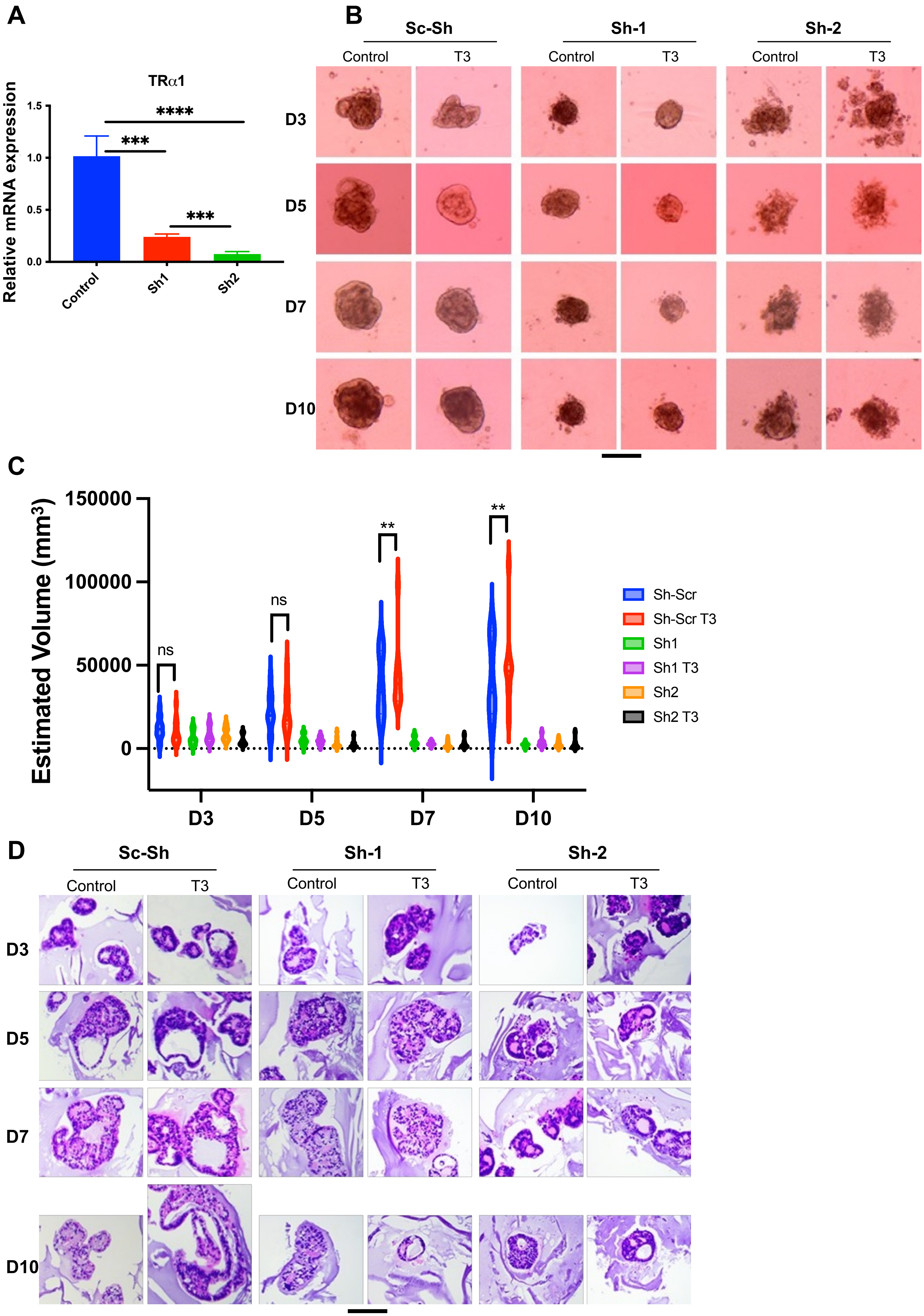

Giolito et al., Figure S3

A

Upregulated genes

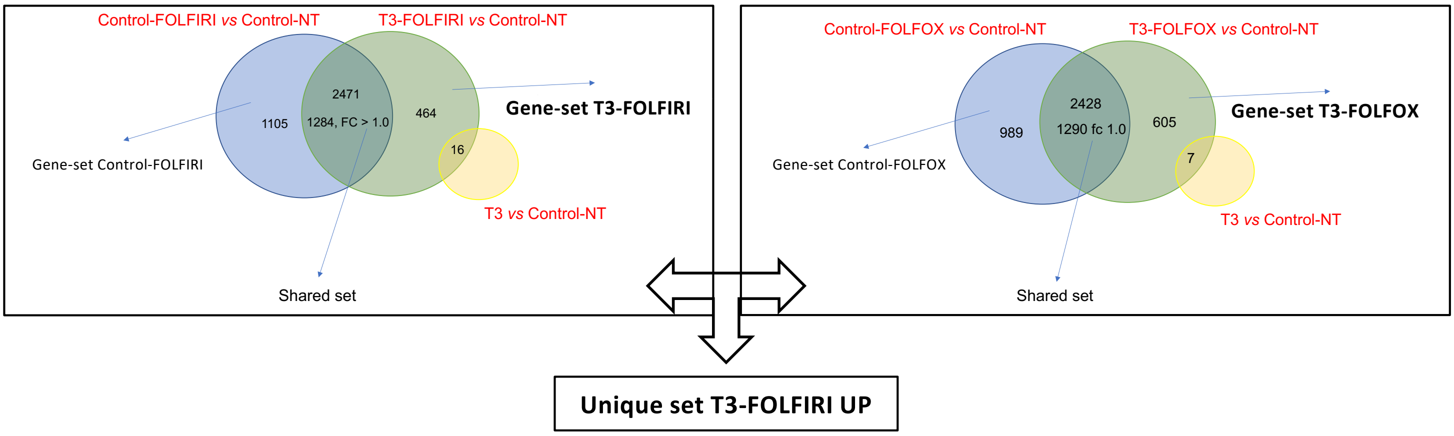

B

Downregulated genes

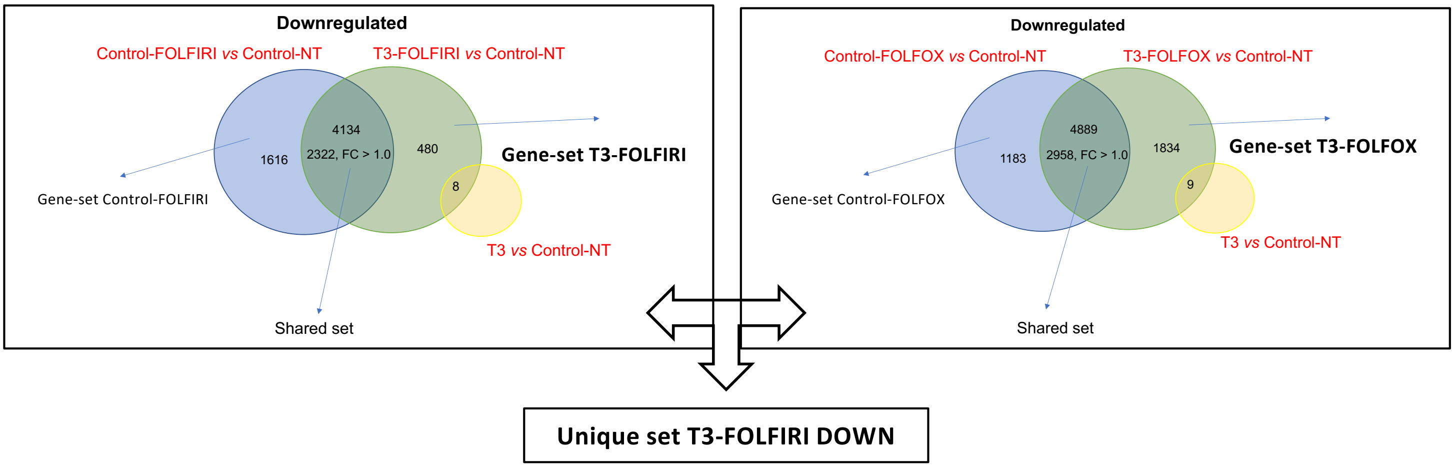

Giolito et al., Figure S4

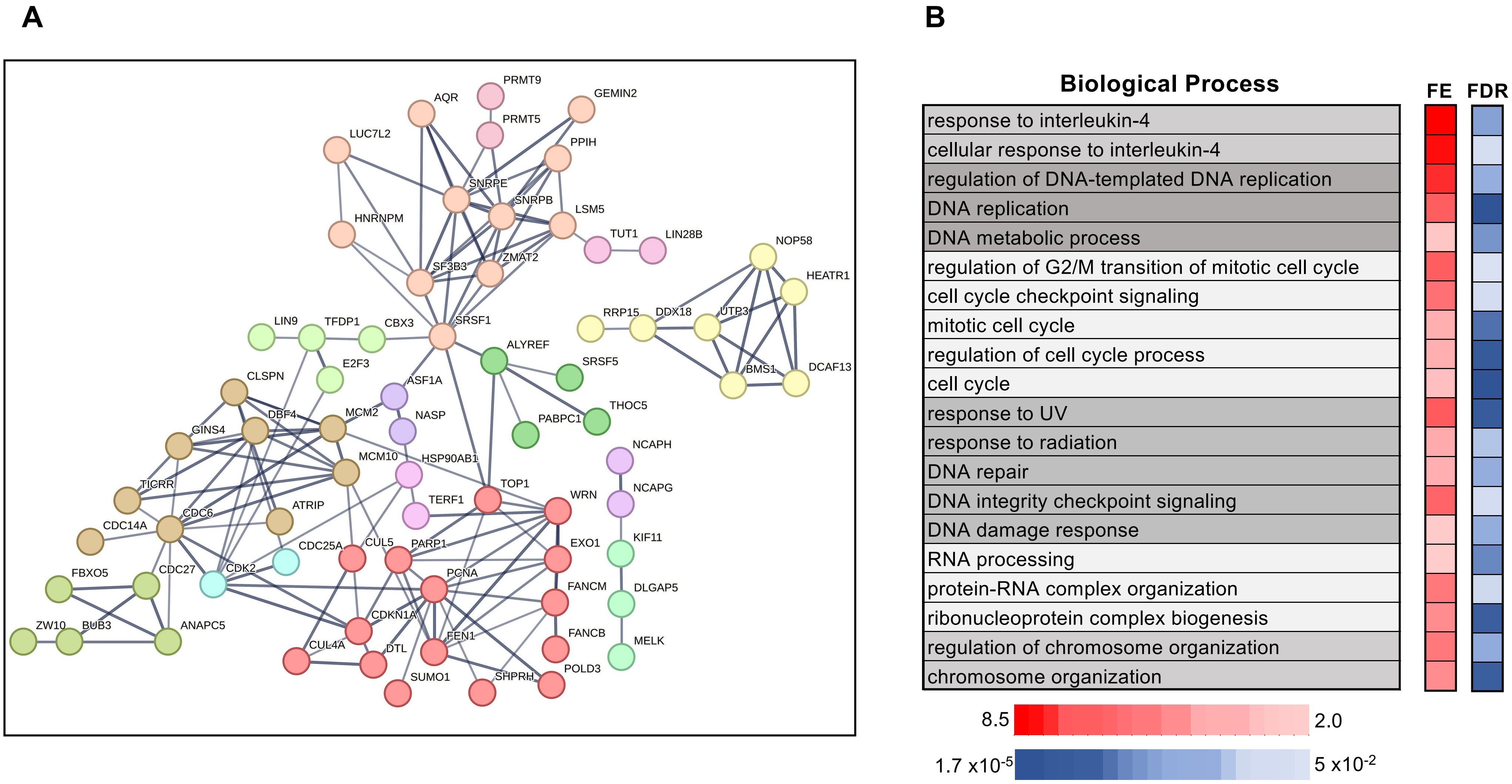

Giolito et al., Figure S5

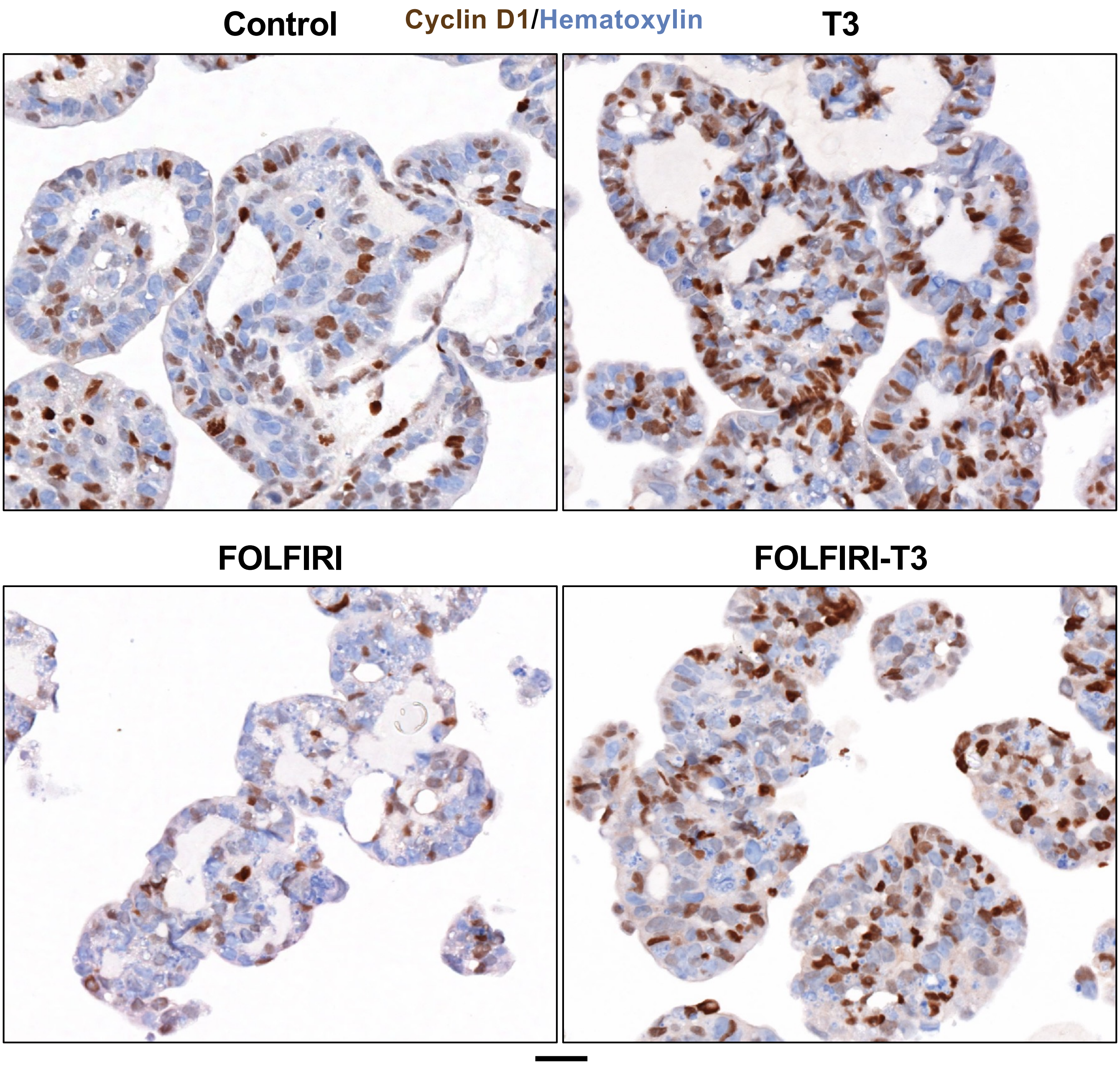

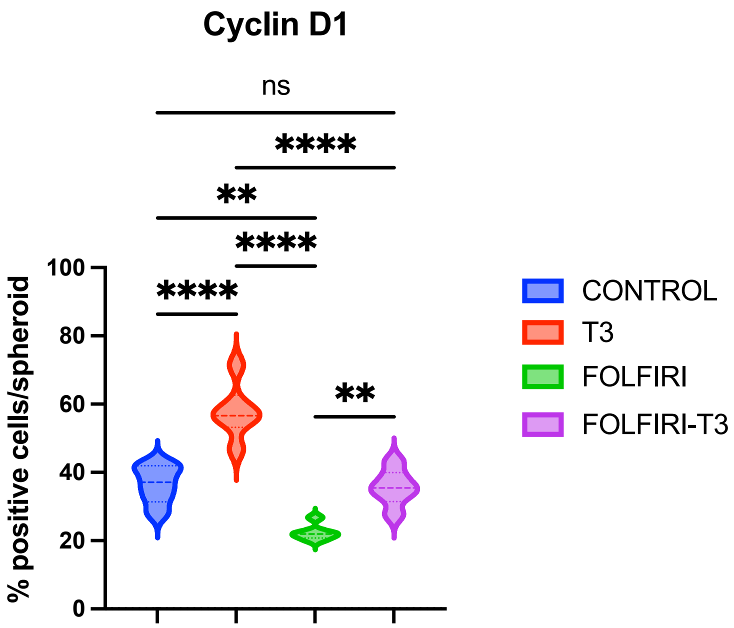

Giolito et al., Figure S6

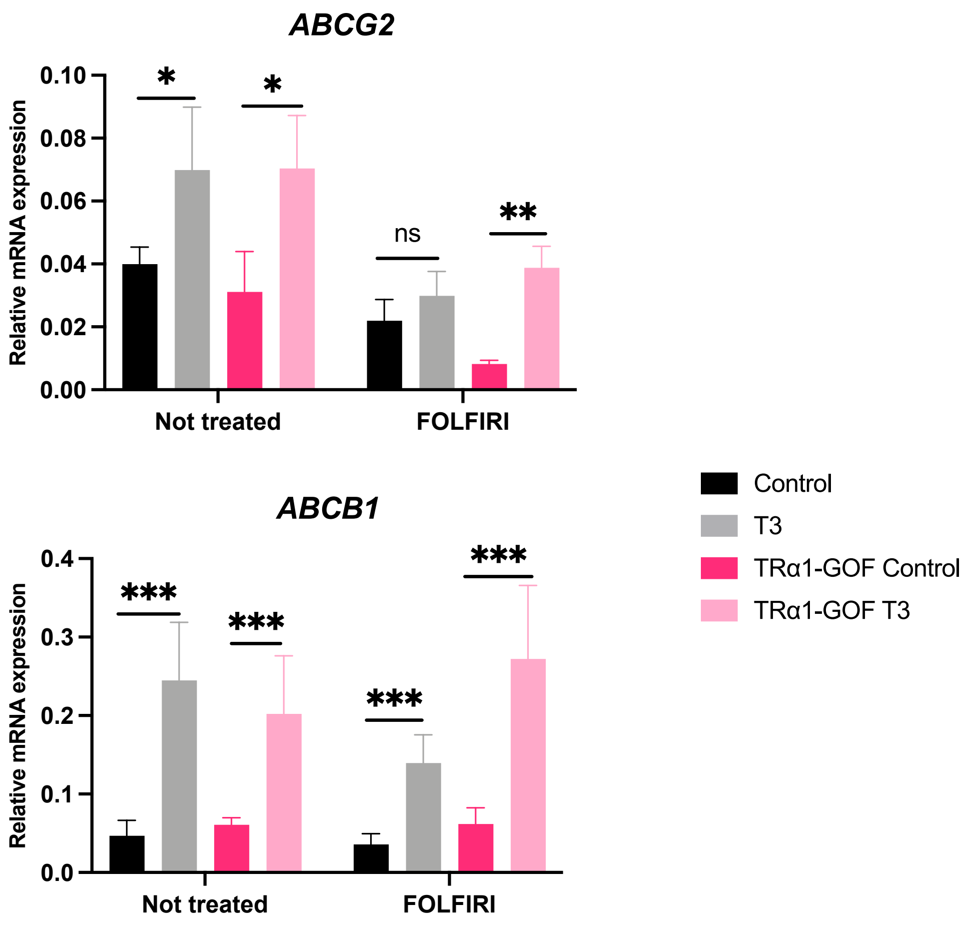

Giolito et al., Figure S7

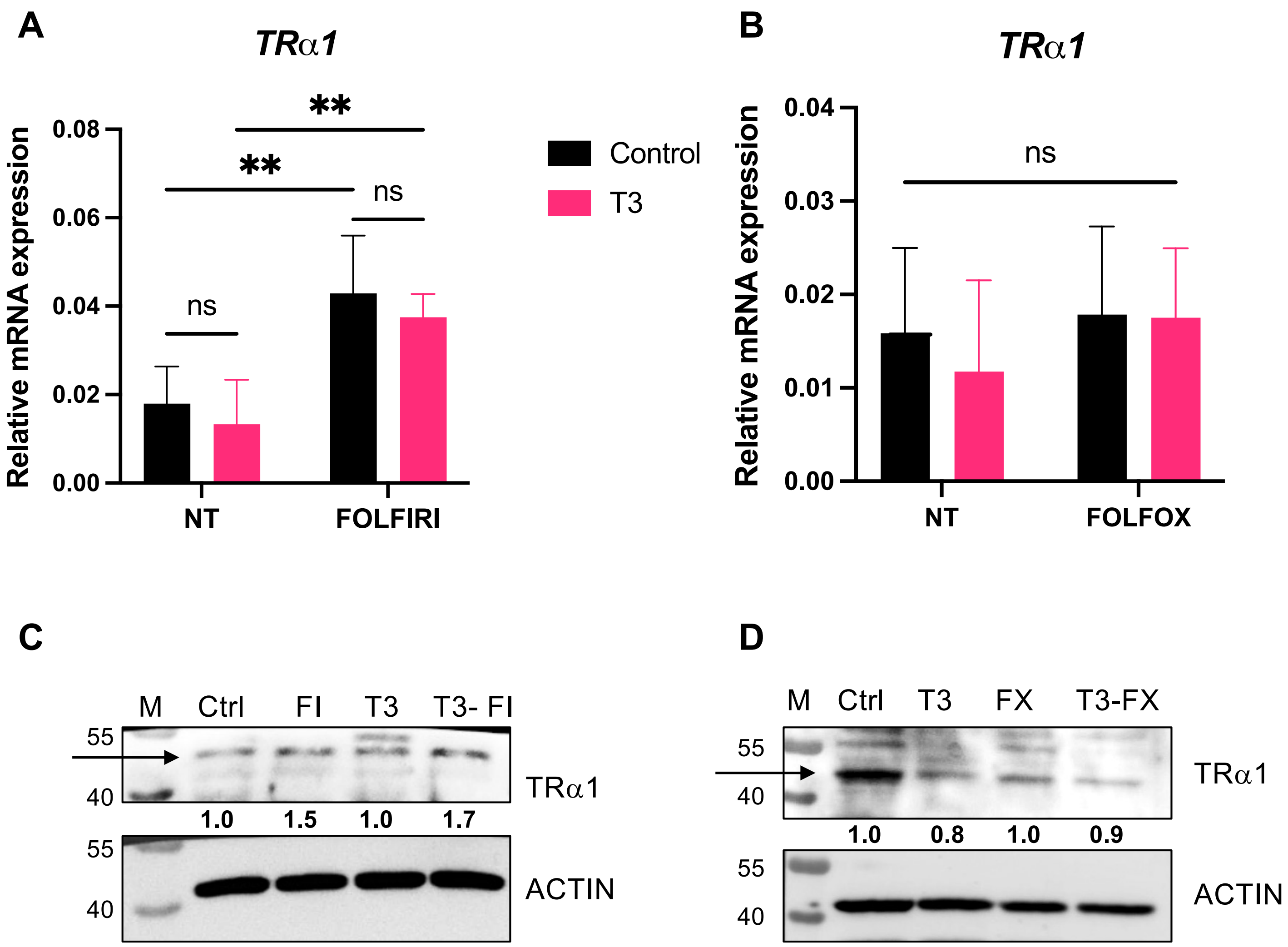

Giolito et al., Figure S8

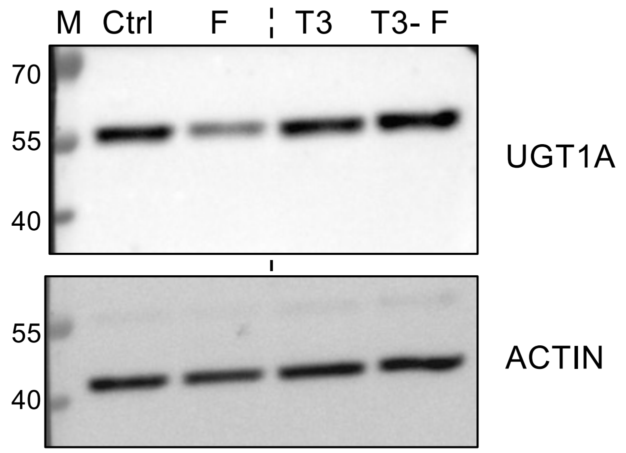

Supplement: Supplementary file 1 — Supplementary Informations Merged [file 41419_2024_6690_MOESM1_ESM.pdf]
