# Supplementary material for: Impact of the thyroid hormone T3 and its nuclear receptor TRα1 on colon cancer stem cell phenotypes and response to chemotherapies
Source: Cell Death Dis. 2024 May 1;15(5):306. doi: 10.1038/s41419-024-06690-x (PMC11063186; doi:10.1038/s41419-024-06690-x)

Giolito et al., Figure S9. Originals WB Figure 8

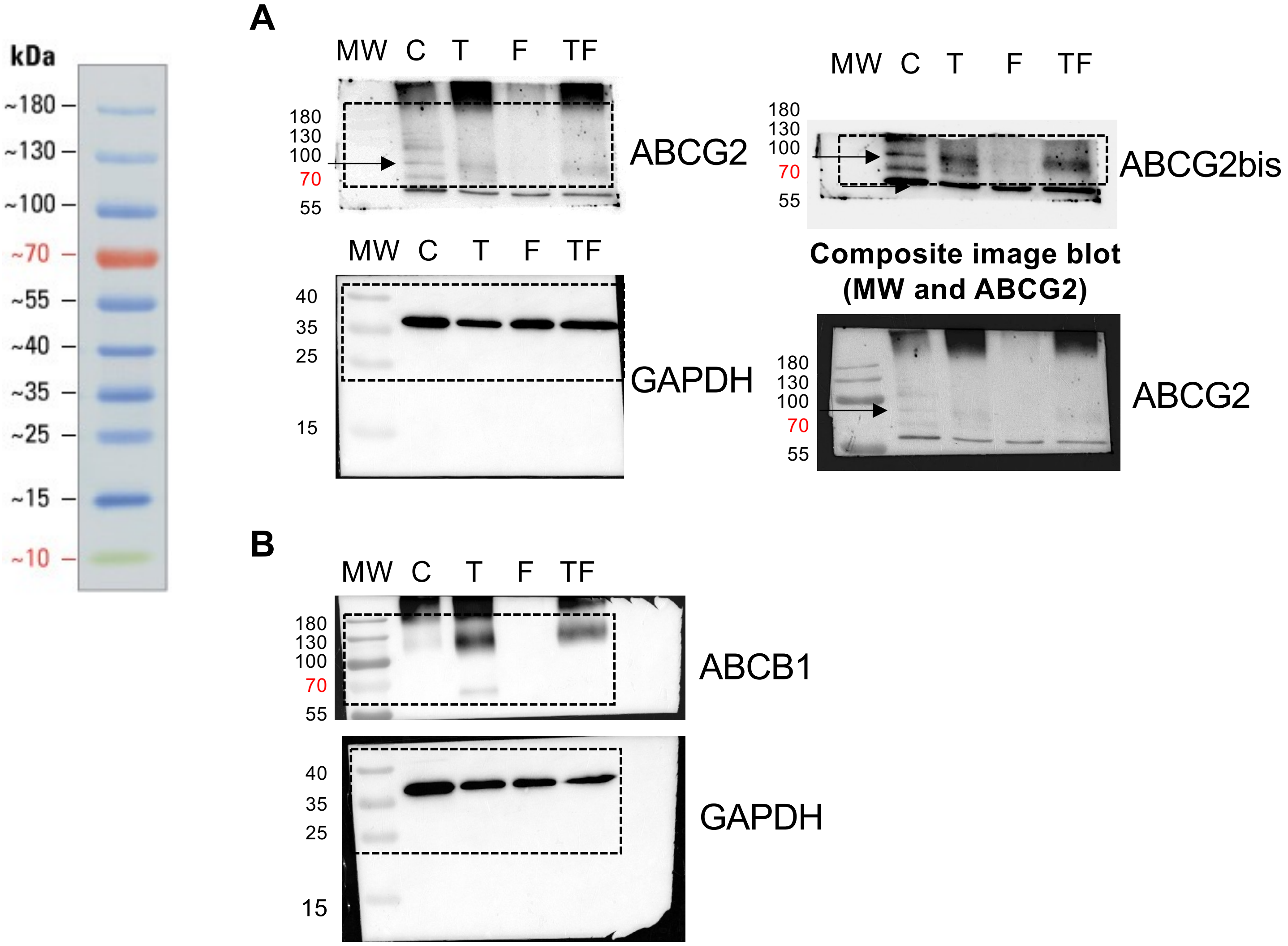

Giolito et al., Figure S10. Originals WB Figure S7

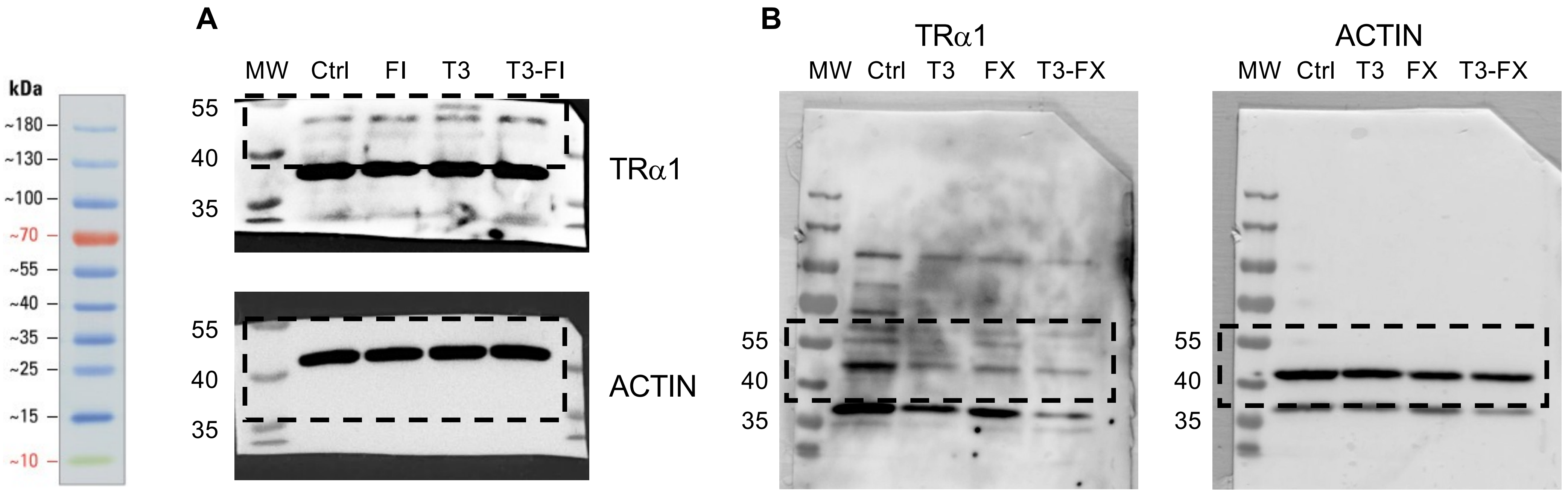

Giolito et al., Figure S11. Originals WB Figure S8

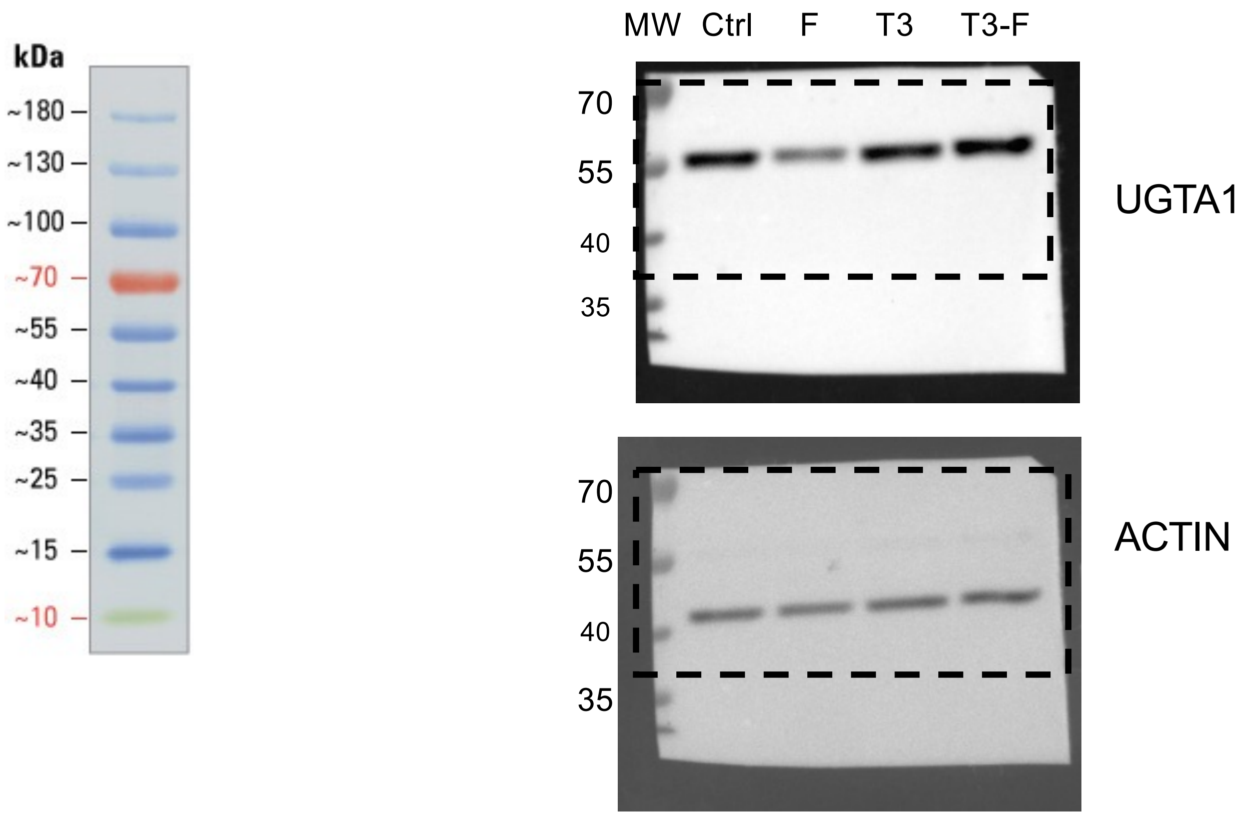

Supplement: Supplementary file 2 — Original Data WBs [file 41419_2024_6690_MOESM2_ESM.pdf]
