# Supplementary material for: Impact of the thyroid hormone T3 and its nuclear receptor TRα1 on colon cancer stem cell phenotypes and response to chemotherapies
Source: Cell Death Dis. 2024 May 1;15(5):306. doi: 10.1038/s41419-024-06690-x (PMC11063186; doi:10.1038/s41419-024-06690-x)
Supplement: Supplementary file 3 — Table S1 and S2 [file 41419_2024_6690_MOESM3_ESM.pdf]

Table S1. Oligos sequences used for RTqPCR and Sh sequences

Gene		Sequence	Product length
PPIB	**F**	ATG ATC CAG GGC GGA GAC TT	100
	**R**	GCC CGT AGT GCT TCA GTT TG	
TRa1	**F**	TGC CTT TAA CCT GGA TGA CAC	240
	**R**	TCG ACT TTC ATG TGG AGG AAG	
ABCG2	**F**	GTG GTG TGT CTG GAG GAG AAA	189
	**R**	TAT CGA GGC TGA TGA ATG GAG	
ABCB1	**F**	AAA CAC CAC TGG AGC ATT GAC	116
	**R**	ATT CCT GTC CCA AGA TTT GCT	

Sh-RNA sequences against TRa1

	Sequence
Sh1	TTGAGGATCAGGAAGTCTAAA
Sh2	TAGTCATTCTAACTGCACTTT

Table S2. Antibodies used for immunolabeling and wester blot

Antibody	Species	Brand	Application	Reference	Dilution
PCNA	mouse	Dako	IF	M0879	1/1000
cleaved-caspase 3 (D175)	rabbit	Cell signalling	IF	9661	1/100
ALDH1A1 (D9J7R)	rabbit	Cell signalling	IF	36671	1/400
ABCG2 (BXP-21)	mouse	Santa Cruz Biotechnology	IF	sc-58222	1/200
ABCB1 (E1Y7B)	rabbit	Cell signalling	IF	#13063	1/800
AlexaFluor 568 anti mouse	donkey	ThermoFisher	IF	A10037	1/1000
AlexaFluor 488 anti rabbit	donkey	ThermoFisher	IF	A121206	1/1000
Ki67	rabbit	Spring Bioscience	IHC	5298512001	1/500
Cyclin D1	rabbit	EmergoEurope	IHC	RMAB003	1/200
ABCG2	rabbit	Gentex	WB	GTX100437	1/1000
ABCB1	rabbit	Cell signaling	WB	13063	1/1000
TRa1	rabbit	Abcam	WB	ab53729	1/500
UGT1A	mouse	SantaCruz	WB	sc-271268	1/500
GAPDH	mouse	SantaCruz	WB	sc-32233	1/1000
ACTIN	mouse	Sigma	WB	A5441	1/10000
Anti-Mouse-HRP	goat	Promega	WB	W402B	1/10000
Anti-Rabbit-HRP	goat	Promega	WB	W401B	1/10000
